# AI in Cancer Prognosis: A Systematic Review of Multimodal Models Combining Pathology Images and High-Throughput Omics

**DOI:** 10.1177/11769351261434523

**Published:** 2026-05-11

**Authors:** Charlotte Jennings, Andrew Broad, Lucy Godson, Emily Clarke, David Westhead, Darren Treanor

**Affiliations:** 1 National Pathology Imaging Cooperative, Leeds Teaching Hospitals NHS Trust, UK; 2 University of Leeds, UK; 3 Department of Clinical Pathology, Linköping University, Sweden; 4 Centre for Medical Image Science and Visualisation, Linköping University, Sweden

**Keywords:** multimodal machine learning, deep learning, artificial intelligence, survival prediction, prognostic models, cancer, histopathology, whole slide images, omics, systematic review, clinical utility, reporting standards

## Abstract

This study systematically reviews and evaluates published research on machine learning models that integrate histopathology whole slide images and high-throughput -omic data to predict overall survival in cancer. A comprehensive search of PubMed, EMBASE, and Cochrane CENTRAL was conducted through August 12, 2024, with citation screening for additional studies. Eligible studies applied machine learning or deep learning methods to multimodal data combining pathology images and -omics. Data extraction followed the CHARMS checklist, and risk of bias was assessed using the PROBAST + AI tool. Narrative synthesis was conducted in line with PRISMA 2020 guidelines. Forty-eight studies published since 2017 met inclusion criteria, spanning 19 cancer types. All relied on The Cancer Genome Atlas dataset. Modelling approaches included regularised Cox regression (n = 4), classical machine learning (n = 13), and deep learning (n = 31). Reported concordance indices ranged from 0.550 to 0.857, with most multimodal models outperforming unimodal counterparts. However, all studies were assessed as having high or unclear risk of bias—most often due to limited external validation, insufficient reporting, and minimal assessment of clinical utility. This review highlights a rapidly evolving yet methodologically underdeveloped field. While model performance is promising, improvements in data standardisation, reporting practices, and real-world contextualisation are critical for clinical translation. This work was funded by the National Pathology Imaging Cooperative (NPIC), supported by UK Research and Innovation (Project no. 104687).

## Introduction

Cancer remains a key health priority globally, with an estimated 18.1 million new cases of cancer worldwide in 2020 and a projected increase to an annual incidence of 28 million by 2040.^1^ Prognostic models that accurately stratify patients by survival risk are essential for guiding treatment decisions, clinical trial design, and for health resource planning making them valuable to patients, clinicians, researchers and policy makers.^2,3^

Routine cancer care involves the integration of multi-modal data—including clinical, radiological, pathological, and molecular inputs—to guide decision making. Digitisation of pathology slides into whole slide images (WSI) and advances in -omics now enable the computational analysis of these data, supporting precision oncology. Clinical prediction models are tools that estimate a patient’s risk of an outcome by combining multiple covariates and have traditionally relied on regression-based methods. With the increasing scale and complexity of multi-modal cancer data available, there has been increasing interest in applying machine learning (ML) and deep learning (DL) approaches to build more flexible, data-driven prognostic models.^4^

ML models have already demonstrated improved survival prediction performance in single-modality settings, including WSI data,^5,6^ and multiomics.^7^ More recent studies report enhanced prognostic predictions by the integration of these data, assumed to be a result of capturing complementary biological signals. To date, clinical translation of ML approaches in other domains has been very low.^8^ There is a need to understand the maturity of this field, whether there is the potential for real-world utility and if the performance gain of these models is sufficient to justify the high costs of laboratory data generation and computation.

In related work, Schneider et al. evaluated studies integrating pathology image and genomic data by DL (searches up to June 2021).^9^ This review identified 11 studies, including 7 predictive of survival (4 cancer-specific survival, 3 overall survival) and 4 related to other tasks, including; prediction of microsatellite instability (MSI) status, malignant versus benign differentiation and subtype prediction. The multimodal approach was shown to be superior to unimodal methods in all studies, however the heterogeneity of studies limited further conclusions. A more general review by Cui et al. across the medical domain also determined that multimodal DL prediction models typically surpass unimodal models for disease diagnosis and prognosis tasks.^10^ This selective review evaluated 34 studies using feature-level multimodal DL-based fusion to integrate image and non-image data, and outlined emerging multimodal frameworks and fusion approaches. Data availability and lack of explainability were identified as key limitations to these studies.

This review provides the first focussed review on prognostic prediction in the cancer domain through high-throughput -omic and WSI integration. In line with best practice methodology, we systematically review all literature in which these data are combined as predictors for the prediction of overall survival (OS) in cancer patients using ML or DL techniques. We focussed on OS as a universally applicable, clinically meaningful outcome that is consistently reported across cancer types and public datasets, enabling standardised comparison across studies. While OS reflects factors beyond tumour biology, its broad availability and relevance made it the most practical and informative endpoint for this review.

This review specifically addresses the following questions;

- What is the prediction performance for OS?
- What cancer domains have these techniques been applied to?
- What types and scale of data sources are used?
- What methods are used for data processing and integration of data?
- What is the quality of these studies and their reporting?

## Methods

This systematic review without meta-analysis was conducted in accordance with the guidelines for “Preferred Reporting Items for Systematic Reviews and Meta-Analyses” (PRISMA).^11^ Due to the highly heterogenous nature of included studies a narrative synthesis approach was used in line with the “Synthesis without meta-analysis” (SWiM) guidelines.^12^ The protocol for this review is available at https://www.crd.york.ac.uk/PROSPERO/display_record.php?RecordID=594745 and was approved before the search results were screened for inclusion (Registration CRD42024594745).

### Eligibility Criteria

Primary, peer-reviewed research studies reporting the development and/or validation of a prediction model developed using ML methods and integrating pathology WSI data with high-throughput -omic data were sought. Only peer-reviewed journal or conference papers were included.

The population included human participants with any cancer diagnosis. The outcome of interest was OS predicted at any time point. Predictive models for other outcomes, including cancer-specific survival, were excluded. The search strategy was geared towards selection of typical ML or DL approaches. However, to recognise ML methodologies which build on traditional statistical models, studies using regression approaches were accepted as ML models if they were defined as such by the authors of a study—an approach used previously.^8,13^ Studies focussing solely on prognostic factor identification or lacking image and -omic data integration were excluded. Only WSIs of surgical pathology specimens with conventional haematoxylin and eosin (H&E) or immunohistochemical staining were included, and studies relating to cytology, forensic or other material were not included, nor were studies using other imaging techniques. Studies in which all models also integrated additional radiology imaging were not included.

Studies not in English were excluded. No exclusions were applied for date of publication.

### Data Sources and Search Strategy

Searches were conducted in 2 research databases (EMBASE and PubMed) and 1 trial registry (Cochrane Central Register of Controlled Trials (CENTRAL)) from inception to 12th August 2024. Searches were restricted to human studies and English language.

The search strategy was composed of terms related to pathology whole slide images, -omic data, machine learning, and cancer. The final search was informed by piloting and preliminary scoping searches using alternative terms and combinations, which were appraised against known key publications in the field. For each category multiple terms were combined with the *OR* operator, before combining categories with the *AND* operator. The full search strategy is available in Supplemental material. Citation checking was also conducted (October 2024) using the selected studies from the abstract screening processes as seed references in a forward and back seed approach using *citation chaser shiny app* as a complementary strategy to address any indexing limitations.^14,15^

### Study Selection

All studies from the literature searches were imported to the *Rayyan* software to manage the screening stages of the review across the team.^16^ First, duplicates were manually removed by C.J. supported by the duplicate detection tool within *Rayyan*. Studies were then screened according to predefined algorithms. One investigator (C.J.) screened all titles and abstracts, with a second independent screen performed by either E.C. or L.G. Disagreements were resolved by discussion with the third investigator. Full text articles were screened in the same way. The screening algorithms are available in the Supplemental material.

### Data Extraction

Data extraction for each study was performed independently by 2 reviewers using a predefined data extraction spreadsheet, which was adapted from a previously developed template.^17^ The template was designed with reference to the “Critical Appraisal and Data Extraction for Systematic Reviews of Prediction Modelling Studies” (CHARMS) checklist and “Prediction model Risk OF Bias ASsessment Tool” (PROBAST) and was updated to align with PROBAST + AI on its release.^18-20^ Additional fields specific to the genomic, digital pathology and multimodal ML research domains were added by the authors, for example data processing details and fusion methods. C.J. reviewed all studies, while the second independent review was performed by either A.B. or L.G. Disagreements were resolved by discussion with the third investigator.

Study level information extracted from papers, included: study demographics, cancer domain, inclusion and exclusion criteria, dataset details and participant characteristics. Models meeting the inclusion criteria within these studies were appraised. Detailed information was extracted as below for the best performing OS prediction model in each study meeting the inclusion criteria (defined as our model of interest), with information about any additional models in the paper extracted in a summarised format. Model specific data fields included: approaches to feature generation and selection, model architecture and fusion approaches, and performance measures. Several fields were added or clarified during data extraction with the agreement of all researchers. Any changes were retroactively applied to all previously extracted studies. The final data extraction template is summarised in Supplemental Material.

Information was sought from the full-text articles, as well as Supplemental Materials where appropriate. Inferences were only made where both researchers were confident to do so and labelled as *unclear* where this was not possible. The well-known nature of The Cancer Genome Atlas (TCGA) dataset meant we were able to deduce some characteristics of the data used even when not explicitly provided, such as H&E staining and fixation characteristics. In these situations, assumed data is marked by an asterisk to help understand completeness of reporting of these fields.

#### Risk of Bias Assessment

The consensus developed PROBAST + AI tool was used to assess the models of interest in this study, providing structured assessment of methodological quality, risk of bias and applicability.^20^ The tool assesses the likelihood of results being affected by the study design, conduct or analysis. Reporting quality was considered within the interpretation of these assessments; however, formal application of reporting checklists (eg, TRIPOD-AI) was outside the scope of this review. The model development and evaluation process are assessed by signalling questions across 4 domains (participants, predictors, outcome and analysis), which probe issues including appropriate cohort selection, predictor definition and measurement, outcome clarity, handling of missing data, overfitting and validation methods. Through these questions reviewers are guided towards judging whether each domain is of high, low or unclear concern for quality and risk of bias. Unclear ratings are used where there is not enough information to make a full assessment. The overall quality and risk of bias ratings are determined by the worst domain score. An overall low-concern rating requires low scores in every domain, whereas a high-concern rating in a single domain will lead to high-concern overall.

The PROBAST + AI tool also assesses the applicability of prediction models to the specific criteria of the systematic review across the predictor, participant and outcome domains. Studies are also determined to be of low concern or high concern, or unclear where there is not enough information to make a full assessment.

Owing to the potential subjective interpretation of the signalling questions in the PROBAST + AI tool, 2 independent researchers completed this process for each model, with disagreements resolved as previous by the third. Each paper was assessed by a pathologist and a computer scientist.

### Data Synthesis

The results of the literature search and screening process were summarised in a PRISMA flow chart generated by *PRISMA Flow Diagram Shiny app*.^21^ All extracted data were summarised in 2 tables, covering study level and model characteristics.

The data synthesis did not include any meta-analysis due to the diversity of the methods used and was designed with reference to the SWiM protocol.^12^ For model characteristics, studies were grouped by the modelling method used and further ordered by year of publication to enable appreciation of any method shift over time. The concordance-index (c-index) was chosen as the comparator metric between studies because it was anticipated to be the most consistently reported evaluation metric based on preliminary searches. Two graphs were generated using the c-index, where it was available, to show (a) comparison of performance between the multimodal model of interest and unimodal model performances explored in the study, and (b) performance variation across different cancer types. Area under the curve (AUC) and time-specific AUC values were also extracted where available. Summary metrics used for calibration, overall performance and clinical utility were also tabulated.

The results of the PROBAST + AI assessments were tabulated by study and presented as summary graphs for quality, risk of bias and applicability.

## Results

**Figure 1.**
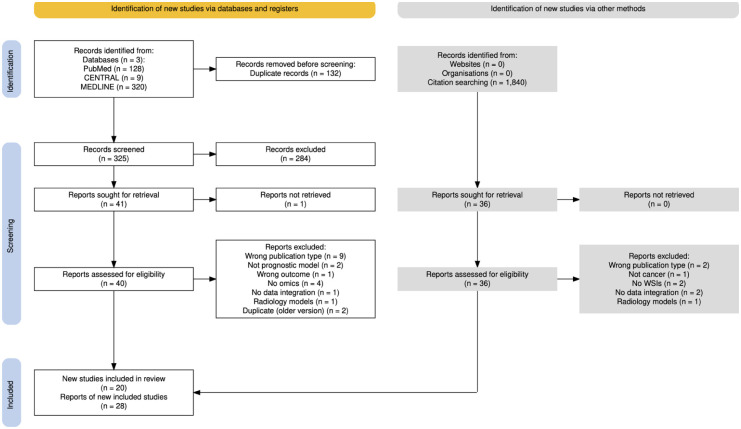
PRISMA 2020 flow diagram. PRISMA 2020 flowchart of the study identification and selection process for the systematic review. Records were screened on titles and abstracts, and reports were assessed based on the full-text content; CENTRAL register of controlled trials.

Literature searches returned a total of 457 studies, of which 132 were duplicates. Two hundred eighty-four records were excluded during abstract and title screening and 21 were excluded by the full text screening process. An additional 1840 titles and abstracts were screened as part of citation searches, of which 36 new studies were screened in full text and 29 included in the review Figure 1. The study characteristics are shown in Table 1. Details of the models evaluated are shown in Table 2.

**Table 1.** Characteristics of the Studies Included in the Systematic Review.

Author, year	Location	Model of interest	Cancer types	Internal data		Ext. data	WSI data	Omic data	Clinical data	Participant characteristics provided in study:			Data available	Code available
				Name	Origin	Name (origin)	Fixation stain resolution	Data types	Variables in model	Number of participants	Demo-graphic information	Disease information		
Boehm et al, 2022^22^	USA	GHC*	(Ovary) HGSOC	MSKCC; TCGA-OV	USA, USA*	-	FFPE H&E -	CNV, somatic mutation, SNP	RD status, PARP inhibitor administration	283 (444)	Age, gender, race	Stage	Partially	Yes
Cheerla and Gevaert, 2019^23^	USA	Multimodal (Clin + miRNA + mRNA + WSI)	(Multi - 20) Pan cancer	TCGA-PanCanAtlas	USA*	-	- H&E -	mRNA, miRNA	Race, age, gender, grade	11 160	-	-	All open source*	Yes
Chen et al, 2021^24^	China	HTRS	(Head & Neck) SCC	TCGA	-	*	- H&E 20x, 40x	mRNA	-	212	Age, gender	Tumour site, grade, stage	On request	-
Chen et al, 2021^25^	China	Multiomics model	(Lung) Adenocarcinoma	TCGA (LUAD)	USA*	*	FFPE H&E 40x	mRNA, genomics, protein expression	-	470	Age, gender	Stage	All open source	-
Chen et al, 2022^26^	USA	MMF	(Multi - 14) Pan cancer	TCGA (BLCA, BRCA, COADREAD, HNSC, KIRC, KIRP, LGG, LIHC, LUAD, LUSC, PAAD, SKCM, STAD, UCEC)	USA*	-	FFPE H&E -	CNV, somatic mutation, mRNA	-	5720	Age, gender, ethnicity	Grade	All open source	Yes
Chen et al, 2022^27^	USA	Pathomic Fusion	(Multi - 2) Glioma	TCGA (LGG, GBM, KIRC)	USA*	-	FFPE* H&E -	CNV, somatic mutation, mRNA	-	769 (1186)	-	-	All open source*	Yes
Cheng et al, 2017^28^	China	Lasso-Cox	Renal (CCRCC)	TCGA	USA*	-	- H&E 20x, 40x	mRNA	-	410	Age, gender	Grade, Stage	Yes	Yes*
Hao et al, 2020^29^	USA	PAGE-NET	(Brain) Glioblastoma	TCGA-GBM	USA*	-	Frozen H&E* 20x	mRNA	Age	447	Age	-	All open source*	Yes
Hou et al, 2022^30^	China	Multi-modality	(Liver) HCC	TCGA	USA*	-	FFPE H&E 5-40x	mRNA	Age, Sex	346	Age, Gender	T group, N group, M group and overall TNM stage	All open source	Yes
Hou et al, 2023^31^	China	HGCN	(Multi - 6) KIRC	TCGA	USA*	-	FFPE* H&E* -	“Genomic profile data”	Age, Gender, Therapy, BMI	385 (2102)	-	-	All open source*	Yes
Ji et al, 2024^32^	China	HGRS	(Renal) CCRCC	TCGA	USA*	-	FFPE H&E 20x, 40x	mRNA	-	281	Age, gender	TNM stage, Grade	All open source	-
Li et al, 2020^33^	China	DeepHit	(Breast) -	TCGA-BRCA	USA*	-	- H&E* -	mRNA	-	826	-	-	All open source	-
Li et al, 2021^34^	China	HGPF	(Colon) Adenocarcinoma	TCGA-COAD	USA*	-	FFPE H&E* -	mRNA	-	199	Age, gender	Stage/TNM classification	All open source	-
Li et al, 2022^35^	China	HFBSurv	(Multi - s10) Breast	TCGA	USA*	-	- H&E 40x	mRNA, CNV	-	1015 (-)	-	-	All open source*	Yes
Liu et al, 2023^36^	China	MGCT	(Multi - 5) Pan cancer	TCGA (LUAD, BRCA, BLCA, GBMLGG, UCEC)	USA*	-	- H&E* -	CNV, somatic mutation, mRNA	-	3557	-	-	All open source*	Yes
Liu et al, 2024^37^	China	IntraSA-InterCA	(Breast) -	TCGA	USA*	-	- H&E* 40x	mRNA	-	345	-	-	All open source*	-
Lv et al, 2021^38^	China	PG-TFNet	(Colorectum) -	TCGA	USA*	-	- H&E -	mRNA	Age, gender, pathologic stage	522	-	-	All open source*	-
Lv et al, 2023^39^	China	Trans-Surv	(Colorectum) -	TCGA; (NCT-CRC-HE-100K; CRC-VAL-HE-7K tissue classifier used in model)	USA*	-	- H&E -	CNV, mRNA	Age, gender, pathologic stage	520	-	-	All open source*	-
Ning et al, 2020^40^	China	Gene + His*	(Renal) CCRCC	TCGA	USA*	-	- H&E* -	mRNA	-	209	Age, gender	Grade, stage	All open source*	Yes
Ning et al, 2023^41^	China	McLR Framework	(Multi - 33) LIHC	TCGA (KIRC, LIHC, LUAD)	USA*	-	- H&E -	mRNA	-	334 (1177)	Age, gender	Stage, pT stage	All open source*	Yes*
Perez-Herrera et al, 2024^42^	Spain	Model 3	(Breast) -	TCGA (BRCA) & TIGER; (segmentation training only); METABRIC	USA*; Netherlands & Belgium	METABRIC (Canada-UK*)	- H&E -	SNP, CNV, mRNA	Age, subtype	3593	Age	Subtype	All open source*	-
Qiu et al, 2024^43^	China	DDM-net	(Brain) Glioma	TCGA (GBMLGG)	USA*	-	FFPE H&E -	CNV, somatic mutation, mRNA	-	954	-	WHO grading	All open source	-
Shao et al, 2020^44^	China	OMMFS	(Multi - 3) LUSC	TCGA (KIRC, KIRP, LUSC)	USA*	-	- H&E* -	CNV, DNAm, mRNA	-	365 (787)	-	Stage	All open source*	-
Shao et al, 2023^45^	China	FAM3L	(Multi - 3) Multi (KIRP)	TCGA (BRCA, KIRP, KIRC)	USA*	-	- H&E* -	mRNA	-	251 (1824)	Age	-	All open source*	-
Shao et al, 2020^46^	China	M2DP	(Multi - 3) BRCA	TCGA (LUSC, BRCA, LIHC)	USA*	-	- H&E* -	mRNA	Stage	790 (1324)	-	Stage	All open source*	-
Shao et al, 2023^47^	China	IMO-TILS	(Multi - 3) Breast	TCGA	USA*	-	- H&E* -	mRNA, miRNA	-	365 (920)	Age	-	All open source*	-
Steyaert et al, 2023^48^	USA	Late fusion	(Brain - Multi) Adult Glioblastoma	TCGA; Paediatric Brain Tumour Atlas (PBTA) Kids First cohort	USA*	CPTAC-GBM (USA*)	FFPE H&E* -	mRNA	-	783 (1088)	-	Low grade, high grade	All open source	Yes
Subramanian et al, 2021^49^	USA	GCCA	(Breast) -	TCGA (BRCA)	USA*	-	- H&E* -	mRNA	-	974	-	-	All open source	Yes
Subramanian et al, 2024^50^	USA	SCCA	(Breast) -	TCGA (BRCA)	USA*	-	- H&E -	mRNA	-	974	Age, gender, ethnicity	-	Yes	Yes
Sun et al, 2018^51^	China	GPMKL	(Breast) -	TCGA (BRCA)	USA*	-	- H&E 40x	CNV, DNAm, mRNA protein expression	-	578	Age	-	All open source*	-
Tan et al, 2022^52^	China	MultiCoFusion	(Brain) GBMLGG	TCGA (GBM, LGG)	USA*	-	- H&E -	mRNA	Grade	469	-	Grade	All open source*	-
Vale-Silva et al, 2021^53^	Germany	MultiSurv	(Multi - 33) Multi	TCGA	USA*	-	- H&E -	CNV, DNAm, mRNA, miRNA, DNAm	Age, gender, race, cancer type, tumour stage, prior malignancy, synchronous malignancy, prior treatment, pharmaceutical treatment, radiation treatment.	11 167	-	-	All open source*	Yes
Vollmer et al, 2024^54^	Germany	Random Survival Forest	(Oral) SCC	TCGA (HNSC)	USA*	-	- H&E 40x	mRNA, somatic/germline DNA	Yes, but unclear what	406	Age, gender, race, ethnicity	Stage	All open source	Yes
Wang et al, 2021^55^	China	GPDBN	(Breast) -	TCGA (BRCA)	USA*	-	- H&E* 40x	mRNA	-	1015	-	-	All open source*	Yes
Wang et al, 2023^56^	China	HC-MAE	(Multi - 6) LGG	TCGA (LIHC, BRCA, LUAD, COAD, LGG, STAD)	USA*	-	- H&E* -	DNAm, mRNA, miRNA	-	451 (2420)	-	-	All open source*	Yes
Wei et al, 2023^57^	China	MultiDeepCox-SC	(Stomach) -	TCGA (STAD)	USA*	*	FFPE H&E 20x	mRNA	Age	357	Age, gender, race	Stage, grade	All open source	-
Wu et al, 2023^58^	China	CAMR	(Multi - 3) LGG	TCGA (BRCA, LUSC, LGG)	USA*	-	- H&E* 40x	CNV, mRNA	-	629 (2135)	-	-	All open source*	Yes
Xie et al, 2024^59^	China	GaCaMML	(Stomach)	Ruijin Hospital (Shanghai); GSE54129 (Public Chinese cohort, Ruijin)	China, China	TCGA-LUAD - (USA*)	- H&E 40x	mRNA	-	63 (95)	Age, sex	Histological type, grade, TNM stage, Bormann classification, tumour position	On request	-
Zeng et al, 2020^60^	China	Multi-omics model	(Head & Neck) SCC	TCGA (HNSCC)	USA*	-	- H&E 20x, 40x	mRNA, somatic mutation, protein expression	-	216	Age, gender	Anatomic site, stage	All open source	-
Zeng et al, 2021^61^	China	Multi-omics model	(Ovary) HGSOC	TCGA	USA*	*	- H&E -	Genomics, mRNA, protein expression	-	229	Age	Stage	All open source*	-
Zhan et al, 2021^62^	China	Two-stage Cox-nnet	(Liver) HCC	TCGA	USA*	-	FFPE H&E -	mRNA	-	290	-	-	All open source*	Yes
Zhang et al, 2020^63^	China	HI-MKL	(Brain) Glioblastoma	TCGA (GBM)	USA*	-	- H&E* 20x, 40x	CNV, DNAm, mRNA	-	251	Age, gender	-	All open source*	-
Zhao et al, 2023^64^	China	Ada-RSIS	(Multi - 3) UCEC	TCGA (BLCA, UCEC, LGG)	USA*	TCGA (GBM, UCS; USA*)	- H&E -	mRNA	-	539 (1437)	Age, gender	Stage	All open source*	Yes*
Zheng et al, 2024^65^	USA	FSM	(Lung - Multi) LUAD	TCGA; National Lung Screening Trial (generating features)	USA*	CPTAC (LUAD, LUSC; USA*)	- H&E* -	mRNA	-	444 (1259)	Age, gender, race	Stage	All open source*	Yes
Zhou et al, 2023^66^	Hong Kong	CMTA	(Multi - 5) GBMLGG	TCGA (BLCA, BRCA, GBMLGG, LUAD, UCEC)	USA*	-	- H&E 40x	CNV, mRNA, SNP	-	569 (2831)	-	-	All open source*	Yes
Zhou et al, 2023^67^	USA	Integrative CNN	(Colon) Adenocarcinoma	TCGA (COAD); Wayne State University	USA*, USA	TCGA-READ (USA*)	FFPE H&E -	Somatic mutation, MSI signature	Age, gender, TNM stage, T stage, N, stage, M stage	Unclear	Age, sex	Stage	Yes	Yes
Zhou et al, 2024^68^	Hong Kong	MSEN	(Multi - 5) GBM-LGG	TCGA (BLCA, BRCA, LUAD, UCEC, GBMLGG)	USA*	-	- H&E* -	CNV, Somatic mutation, mRNA	-	492 (2670)	-	-	All open source*	Yes*
Zhu et al, 2023^69^	China	SAMMS	(Multi - 2) LGG	TCGA (LGG, KIRC)	USA*	-	- H&E* 20x	CNV, mRNA, miRNA	Age, Gender	500 (806)	-	-	All open source*	-

**Asterisk** generally used to denote fields where answer reasonably assumed by both investigators; - used where information not provided or unclear; **Model of interest (asterisk)** – indicates where an ineligible multimodal model (eg, incorporating radiology) achieved a higher result than our model of interest; **External data (asterisk)** where external validation datasets are used within the study but not for the multimodal model. **Code availability (asterisk)** – indicates where code links not provided or where link did not lead to associated code despite described as being available.

Abbreviations: BMI, body mass index; CNV, copy number variation; CPTAC, clinical proteomic tumour analysis consortium; DNAm, DNA methylation; FFPE, formalin fixed paraffin embedded; H&E, haematoxylin and eosin; mRNA, messenger RNA; miRNA, micro-RNA; MSKCC, memorial Sloan Kettering cancer centre; SNP, Single nucleotide polymorphisms; TCGA, the cancer genome atlas.

**Table 2.** Characteristics of the Models of Interest Included in the Systematic Review.

Method	Author, year (ref)	Modelling description	Study size	Events	Predictors		WSI Feature	Omic feature	Fusion level	Validation	Performance measures				Internal results			
			n	n (%)	Cand.	Final	Approach (named method)	Named method (s)			Calibration	Discrimination	Overall	Clin. Utility	C-index	Variability (metric)	AUC	Variability (metric)
ML regularised regression	Cheng et al, 2017^28^	**Lasso-Cox** Image features and eigengenes into lasso-regularised Cox Proportional Hazards model	410	135 (33)	165	13	Hand crafted -	lmQCM	Feature	Cross-validation	-	Log-rank test/Risk group curves	-	-	-	-	-	-
	Shao et al, 2020^44^	**OMMFS** Feature selection via ordinal multi-modality feature selection model, then features into Cox Proportional Hazards model	365	145 (40)	2011	16	Hand crafted -	WGCNA	Feature	Random split data	-	C-Statistic/AUC graph/Log-rank test/Risk group curves	-	-	0.773	x	0.793	x
	Shao et al, 2020^46^	**M2DP** Multi-task, multi-modal feature selection algorithm with Cox Proportional Hazards model	790	82 (10)	-	155	Hand crafted -	lmQCM	Feature	Cross-validation	-	C-Statistic/Log-rank test/Risk group curves	Brier score	-	0.726	±0.06 (unclear)	-	-
	Shao et al, 2023^45^	**FAM3L** Feature-aware multi-modal metric learning method learns survival associated features which are combined into Cox Proportional Hazards model	251	38 (15)	-	163	Hand crafted -	lmQCM	Feature	Cross-validation	-	C-Statistic/AUC graph/Log-rank test/Risk group curves	-	-	0.752	±0.061 (unclear)	0.791	0.069 (unclear)
Machine learning	Sun et al, 2018^51^	**GPMKL** Selected features integrated by multiple kernel learning, with a separate kernel per data modality	578	445 (77)	59 428	350	Hand crafted cellprofiler	-	Feature	Cross-validation	-	C-Statistic/AUC graph	-	-	0.643	x	0.828	±0.034 (St Err)
	Zeng et al, 2020^60^	**Multi-omics model** Extracted image and omic features integrated directly into random forest model	216	106 (49)	35 302	954	Hand crafted cellprofiler	-	Feature	Cross-validation	-	AUC graph/Log-rank test/Risk group curves	-	DCA	-	-	0.929 (5-y)	x
	Zhang et al, 2020^63^	**HI-MKL** Histopathological image feature integrating multiple kernel learning method - multiple kernels derived from selected features are combined within each data type, before combining across modalities for a combined kernel for MKL.	251	204 (81)	66 650	350	Hand crafted cellprofiler	-	Feature	Cross-validation	-	AUC graph/Risk group curves	-	-	-	-	0.932	x
	Chen et al, 2021^25^	**Multiomics model** Selected histopathology image and multiomics features (multiple selection techniques) integrated by Random Forest model	470	169 (36)	-	954	Hand crafted cellprofiler	-	Feature	Cross-validation	-	AUC graph/Log-rank test/Risk group curves	-	DCA	-	-	0.938 (3-y)	x
	Chen et al, 2021^24^	**HTRS** Selected histopathology image and gene features integrated by a Random Forest model	212	-	5532	259	Hand crafted cellprofiler	WGCNA	Feature	Cross-validation	Calib. plot	C-Statistic/AUC graph/Log-rank test/Risk group curves	-	DCA	0.768	0.715-0.820 (95%CI)	0.826 (5-y)	x
	Li et al, 2021^34^	**HGPF** Direct integration of features selected by SVM-RFE and LASSO-COX (images), and WGCNA (hub genes) into Random Forest	199	32 (16)	20 344	377	Hand crafted cellprofiler	WGCNA	Feature	Cross-validation	Calib. plot	AUC graph/Log-rank test/Risk group curves	-	DCA	-	-	0.924 (5-y)	x
	Subramanian et al, 2021^49^	**GCCA** Graph-structured variant of sparse canonical correlation analysis, capturing intra and intermodality correlations	974	-	-	3213	Hand crafted cellprofiler	-	Feature	Cross-validation	-	-	F1 score	-	-	-	-	-
	Zeng et al, 2021^61^	**Multi-omics model** Extracted image features and selected omics features integrated directly into Random Forest model	229	133 (58)	-	970	Hand crafted cellprofiler	-	Feature	Cross-validation	-	AUC graph/Log-rank test/Risk group curves	-	DCA	-	-	0.911 (5-y)	x
	Ning et al, 2023^41^	**McLR Framework** Unimodal features concatenated into multimodal feature matrix and used as input to multi-constraint latent representation framework	334	116 (35)	20 003	256	Hand crafted PFTAS	-	Feature	Cross-validation	-	C-Statistic/Log-rank test/Risk group curves	-	-	0.72	0.045 (StD)	-	-
	Zhao et al, 2023^64^	**Ada-RSIS** Adaptive risk-aware sharable and individual subspace learning - subspace learning of modality specific and modality sharable representations passed through function to capture intra and inter modality terms. Representations integrated via grouping co-expression constraint and gaussian-based weighting strategy.	539	91 (17)	20 003	256	Hand crafted PFTAS	-	Feature	Cross-validation	-	C-Statistic/Risk group curves	-	-	0.711	±0.048 (StD)	-	-
	Ji et al, 2024^32^	**HGRS** Direct integration of features selected by SVM-RFE and LASSO-COX (images), and WGCNA (hub genes) into Random Forest survival model	281	76 (27)	-	8	Hand crafted cellprofiler	WGCNA	Hybrid	Cross-validation	Calib. plot	AUC graph/Log-rank test/Risk group curves	-	DCA	-	-	0.780 (5-y)	x
	Subramanian et al, 2024^50^	**SCCA** Probabilistic graph modelling by penalised canonical correlation analysis-based joint embeddings and Cox Proportional Hazards model	974	-	21 075	2075	Hand crafted cellprofiler	-	Feature	Cross-validation	-	C-Statistic/Risk group curves	-	-	0.683	±0.0669 (unclear)	-	-
	Vollmer et al, 2024^54^	**Random Survival Forest** Feature selection via then Random Forest model for survival prediction.	406	177 (44)	-	242	Hand crafted cellprofiler	-	Feature	Cross-validation	-	C-Statistic	-	-	0.834	x	-	-
Deep learning	Cheerla et al, 2019^23^	**Multimodal (Clin + miRNA + mRNA** **+** **WSI)** Modality specific features generated via multiple CNNs compressed by unsupervised encoder into single vector for survival prediction by cox loss function. Multimodal dropout approach used in training.	11 160	-	62 308	2048	Learned SqueezeNet CNN	Deep Highway Neural Network	Feature	Random split data	-	C-Statistic	-	-	0.78	x (average result)	-	-
	Hao et al, 2020^29^	**PAGE-NET** Pathology (CNN), genome and clinical (adapted CoxPASNet) layers fused in Cox Proportional Hazards model	447	-	6405	81	Learned Pre-trained CNN	CoxPASNET	Feature	Cross-validation	-	C-Statistic/Wilcoxon rank-sum test	-	-	0.702	±0.0294 (unclear)	-	-
	Li et al, 2020^33^	**DeepHit** Fully connected neural network with image and omic feature fusion via conditional autoencoder.	826	-	-	-	Learned ResNet, DCGMM	-	Feature	Cross-validation	-	C-Statistic	-	-	0.757	0.713-0.801 (95%CI)	-	-
	Ning et al, 2020^40^	**Gene + His** Cross-modal feature-based integrative framework. Deep features extracted from images (CNNs), combined with eigengenes (WGCNA) and fed into cox model for prediction	209	60 (29)	-	138	Learned CNN	WGCNA	Feature	Cross-validation	-	C-Statistic/Log-rank test/Risk group curves	-	-	0.638	0.527-0.749 (unclear)	-	-
	Lv et al, 2021^38^	**PG-TFNet** Output of transformer-based multiscale pathological feature fusion module (images) and multilayer perceptron features (genes) integrated by cross attention transformer-based multimodal feature fusion module and fed into cox layer (with clinical data) for survival prediction.	522	-	-	-	Learned ResNet (multiscale)	MLP	Feature	Cross-validation	-	C-Statistic/Log-rank test/Risk group curves	-	-	0.816	±0.016 (unclear)	-	-
	Vale-Silva et al, 2021^53^	**MultiSurv** Dedicated deep learning sub models per data modality (n = 6), fed into data fusion layer then final network takes fused feature representation and outputs conditional survival probabilities.	11 081	3601 (32)	108 437	3072	Learned ResNet50 CNN (ImageNet)	CNN	Feature	Bootstrap	-	C-Statistic/Log-rank test/Risk group curves	Brier score	-	0.787	0.769-0.806 (95%CI)	-	-
	Wang et al, 2021^55^	**GPDBM** Outputs of modality specific bilinear feature encoding modules (BFEM) and an inter-modality BFEM fused and fed into fully connected neural network for survival prediction	1015	-	20 446	64	Hand crafted cellprofiler	-	Feature	Cross-validation	-	C-Statistic/AUC graph/Log-rank test/Risk group curves	Sensitivity, specificity, accuracy, precision, F1	-	0.725	±0.066 (unclear)	0.808	±0.053 (unclear)
	Zhan et al, 2021^62^	**Two-stage Cox-nnet** Outputs of modality specific cox-nnet models integrated as nodes to a second stage cox-nnet with an output layer for cox regression.	290	-	-	-	Hand crafted cellprofiler	-	Feature	Cross-validation	-	C-Statistic/Log-rank test/Risk group curves	-	-	0.75	x	-	-
	Boehm et al, 2022^22^	**GHC** Fusion of negative log partial hazards from unimodal cox models with learned image features into a multimodal linear cox proportional hazards model	283	-	-	5	Combo ResNet18	HRD status inferred	Decision	Random split data	-	C-Statistic/Log-rank test/Risk group curves/Kendall rank correlation	-	-	0.55	0.531-0.563 (95%CI)	-	-
	Chen et al, 2022^26^	**MMF** Kronecker product fusion of unimodality subnetworks to generate multimodal representation feature and predict survival via log likelihood function	5720	1651 (29)	-	64	Learned ResNet50 (ImageNet), attention module	SNN	Feature	Cross-validation	-	C-Statistic/AUC graph/Log-rank test/Risk group curves	-	-	0.644	x (average result)	0.662	x (average result)
	Chen et al, 2022^27^	**Pathomic Fusion** Unimodal networks as feature extractors (CNN and GCN - images, Feed-forward SNN - genomic) fused by gating attention mechanism and Kronecker product to model modality interactions. Network supervised by cox partial likelihood loss function.	769	-	-	-	Combo VGG19 (ImageNet), GCN	SNN	Feature	Cross-validation	-	C-Statistic/Log-rank test/Risk group curves	-	-	0.826	±0.009 (unclear)	-	-
	Hou et al, 2022^30^	**Multi-modality** Pathology patch level risk score (via multi-instance fully convolutional network aggregated to patient level by attention mechanism) integrated with hub genes into Cox Proportional Hazards model.	346	123 (36)	5005	8	Learned VGG19	WGCNA	Hybrid	Cross-validation	Calibration plot	C-Statistic/AUC graph/Log-rank test/Risk group curves	-	DCA	0.746	±0.077 (95%CI)	0.816 (1-y)	x
	Li et al, 2022^35^	**HFBSurv** Fusion of modality-specific and cross-modality attentional factorised bilinear modules with cox partial likelihood loss function for survival prediction	1015	-	46 125	240	Hand crafted cellprofiler	-	Feature	Cross-validation	-	C-Statistic/AUC graph/Log-rank test/Risk group curves	-	-	0.766	±0.024 (unclear)	0.806	±0.025 (unclear)
	Tan et al, 2022^52^	**MultiCoFusion** Features concatenated then fused via three-layer FCNN before output fed to multitask output.	469	-	62 437	2000	Learned ResNet-152 (ImageNet)	SGCN	Feature	Cross-validation	-	C-Statistic/AUC graph/Risk group curves	-	-	0.857	±0.015 (unclear)	0.915	±0.016 (unclear)
	Hou et al, 2023^31^	**HGCN** Distinct modality graph representations fed into Graph Convolutional Network layers then hypergraph convolutional layer with hyperedge mixing module (intermodal interaction) before decision via cox loss. Jointly trained with online masked autoencoder (to simulate missing data)	385	-	21 504	3	Learned KimiaNet (CNN)	GSEA	Decision	Cross-validation	-	C-Statistic/Log-rank test/Risk group curves	Brier score	-	0.747	±0.007 (95%CI)	-	-
	Liu et al, 2023^36^	**MGCT** Modality specific embeddings integrated with two-stage mutual guided cross-modality transformer framework	3557	-	-	256	Learned ResNet50 (ImageNet)	SNN	Feature	Cross-validation	-	C-Statistic/AUC graph/Log-rank test/Risk group curves	-	-	0.663	x (average result)	-	-
	Lv et al, 2023^39^	**Trans-Surv** Multi Scale Fusion Transformer (image feature extraction), integration of image and omic data by cross-modal fusion transformer, combined with clinical data into Cox Layer	520	-	-	-	Learned Multiple CNNs	-	Feature	Cross-validation	-	C-Statistic/Log-rank test/Risk group curves	-	-	0.822	±0.023 (unclear)	-	-
	Shao et al, 2023^47^	**IMO-TILS** Deep generalised canonical correlation analysis with attention layer to fuse image and multi-omic data	365	32 (9)	272	272	Learned Unet++, ResNet-101 (ImageNet), KNN	lmQCM	Feature	Cross-validation	-	C-Statistic/AUC graph/Log-rank test/Risk group curves	-	-	0.709	±0.030 (unclear)	0.737	±0.047 (unclear)
	Steyaert et al, 2023^48^	**Late Fusion** Modality specific cox proportional hazards models using model derived image (CNN) and omic (MLP) features. Outputs integrated into a final cox regression module (late fusion)	783	-	12 879	4096	Learned ResNet50 CNN	MLP	Decision	Cross-validation	-	C-Statistic/Log-rank test/Risk group curves	Brier score, Composite score	-	0.797	±0.019 (StD)	-	-
	Wang et al, 2023^56^	**HC-MAE** Hierarchical cross-attention masked autoencoder - multiscale WSI representations (guided by omic cross attention)and omic features fed through separate transformers then fused via concatenation. Survival function negative log partial likelihood.	451	-	-	2052	Learned vision transformer-based masked autoencoder	MLP	Feature	Cross-validation	-	C-Statistic/Log-rank test/Risk group curves	-	-	0.851	±0.033 (unclear)	-	-
	Wei et al, 2023^57^	**MultiDeepCox-SC** Integration of age and gene predictors with risk score of deep learning-based image model (DeepCoxSC) using a cox model	357	142 (40)	61 584	12	Hand crafted cellprofiler	-	Hybrid	Cross-validation	-	C-Statistic/AUC graph/Log-rank test/Risk group curves	-	-	0.744	±0.070 (unclear)	0.833 (2-y)	±0.055 (unclear)
	Wu et al, 2023^58^	**CAMR** Cross-aligned multimodal representation learning - modality specific representations and modality invariant representations learnt by separate networks and fused by a gating attention mechanism and concatenation. Survival function is cox partial likelihood loss.	629	-	-	240	Hand crafted cellprofiler	-	Feature	Cross-validation	-	C-Statistic/AUC graph/Risk group curves	-	-	0.841	±0.20 (unclear)	0.889	±0.017 (unclear)
	Zhou et al, 2023^66^	**CMTA** Cross-modal translation alignment - two parallel encoder-decoder structures integrate intra-modal representations and cross-modal representations generated by an attention mechanism.	569	-	-	-	Learned ResNet-50 (ImageNet)	FC NN	Feature	Cross-validation	-	C-Statistic/Log-rank test/Risk group curves	-	-	0.853	±0.0116 (unclear)	-	-
	Zhou et al, 2023^67^	**Integrative CNN** Tile level CNN image features concatenated with clinical variables and mutation status and/or signature. Final feature vector into MLP to predict risk.	-	-	-	-	Learned Inception V3 (ImageNet)	MSI status	Feature	Cross-validation	-	C-Statistic/AUC graph/Risk group curves	-	-	0.69	±0.19 (unclear)	0.8	x
	Zhu et al, 2023^69^	**SAMMS** Pre-trained SAM for images + omic data into modality specific and cross-modality common subnetworks. Outputs concatenated with clinical data and fusion representation used for cox prediction layer.	500	-	-	102	Learned SAM (pretrained)	-	Feature	Cross-validation	-	C-Statistic/AUC graph/Log-rank test/Risk group curves	-	-	0.843	x	0.782	x
	Liu et al, 2024^37^	**IntraSA-InterCA** Modality specific and intramodality modules for generation of feature embeddings integrated by an adaptive fusion block into a combined feature. Survival prediction via multilayer deep neural network.	345	-	22 779	64	Hand crafted cellprofiler	-	Feature	Cross-validation	-	C-Statistic/AUC graph/Log-rank test/Risk group curves	Specificity, accuracy, sensitivity, precision, F1	-	0.742	±0.039 (unclear)	0.841	±0.032
	Perez-Herrera et al, 2024^42^	**Model 3** Integration of image derived variables (UNet), omic features (multiple models) with clinical features via Cox Proportional Hazards model	3593	-	-	12	Learned -	-	Feature	Cross-validation	-	Hazard ratios	-	-	-	-	-	-
	Qiu et al, 2024^43^	**DDM-Net** Dual-space disentangled-multimodal adversarial autoencoder comprising two visual autoencoders and a fusion module input to a cox proportional hazards model for survival prediction. Two imputation networks designed to handle missing data.	954	-	121 415	2048	Learned ResNet50 (ImageNet)	SNN	Feature	Cross-validation	-	C-Statistic/Log-rank test/Risk group curves	-	-	0.824	±0.10 (unclear)	-	-
	Xie et al, 2024^59^	**GaCaMML** Extracted features fed through cross-modal attention layer and MIL aggregation layer of model before fusion of gene and image features by concatenation. The log-likelihood loss function is used for survival prediction.	63	12 (19)	30 000	256	Learned ResNet50 (ImageNet)	FC NN	Feature	Cross-validation	-	C-Statistic/Log-rank test/Risk group curves	-	-	0.613	x	-	-
	Zheng et al, 2024^65^	**F**SM Undirected graph embeddings combined using an attention-based mechanism	444	156 (35)	-	-	Learned CNN (pretrained)	FC NN	Feature	Cross-validation	-	C-Statistic/AUC graph	-	-	0.703	0.017 (StD)	0.679	0.06 (StD)
	Zhou et al, 2024^68^	**MSEN** Multimodal Survival Ensemble Network - unimodal bag representations of WSIs and omic data are passed through feature encoders and attention-based modules, then integrated via an ensemble strategy and input to Cox Proportional Hazard model	492	-	-	-	Learned HIPT	Ensemble based encoder	Feature	Cross-validation	-	C-Statistic/Risk group curves	-	-	0.827	±0.018 (StD)	-	-

Abbreviations: Calib, calibration; CI, confidence interval; Clin., clinical; CNN, convolutional neural network; DCA, decision curve analysis; DCGMM, deep conditional gaussian mixture model; FC, fully connected; GCN, graph convolutional network; GSEA, gene set enrichment analysis; HIPT, hierarchical image pyramid transformer; HRD, homologous recombination deficiency; KNN, K nearest neighbour; lmQCM, local maximal quasi-clique merger; MLP, multi-layer perceptron; PFTAS, parameter free threshold adjacency statistics; SAM, segment anything model; SGCN, sparse graph convolutional network; SNN, self-normalising network; StD, standard deviation; StErr, standard error; WGCNA, weighted gene co-expression network analysis.

Of the 48 included studies,^22–69^ 10 of these were conference papers with the remaining 38 being journal papers. All included studies were published since 2017. Studies appeared in 31 different publications, mainly in computer science and computational biology focussed journals (n = 22), but also in scientific, medical and medical imaging domains. Eight of the studies were in cancer specific journals. Lead authors were distributed across 5 countries; China (n = 33), USA (n = 10), Germany (n = 2), Hong Kong (n = 2) and Spain (n = 1).

### Risk of Bias Assessments

**Figure 2.**
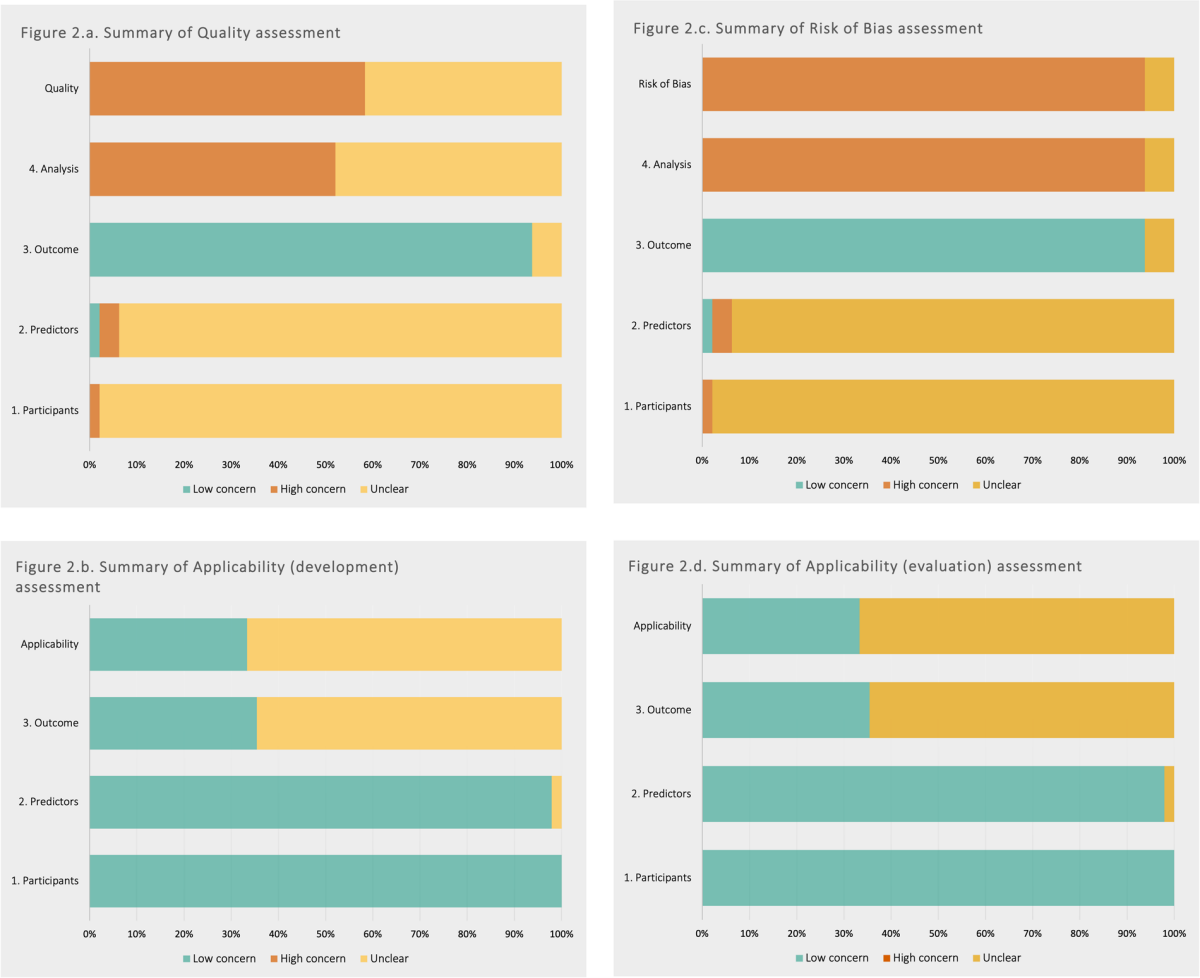
PROBAST + AI summary results. Summarised results for the assessments of (a) quality and (b) applicability (study development), and (c) risk of bias and (d) applicability (study evaluation) for the 48 studies in this review.

The results of the PROBAST + AI assessments for each model of interest are shown in Table 3 and risk of bias and applicability across the review are summarised in Figure 2. Due to the limited use of external datasets for validation, assessments across the participant, predictor and outcome domains for quality of model development and risk of bias of evaluation are usually the same.

**Table 3.** Risk of Bias and Applicability Assessment.

Author, year (ref)	Development							Evaluation							Overall			
	Quality				Applicability			Risk of bias				Applicability			Dev		Eval	
	1. Participants	2. Predictors	3. Outcome	4. Analysis	1. Participants	2. Predictors	3. Outcome	1. Participants	2. Predictors	3. Outcome	4. Analysis	1. Participants	2. Predictors	3. Outcome	Quality	Applicability	Risk of Bias	Applicability
Boehm et al, 2022^22^	?	-	+	?	+	?	+	?	-	+	-	+	?	+	-	?	-	*?*
Cheerla and Gevaert, 2019^23^	?	?	+	?	+	+	+	?	?	+	-	+	+	+	?	+	-	*+*
Chen et al, 2021^24^	?	?	+	?	+	+	+	?	?	+	?	+	+	+	?	+	?	*+*
Chen et al, 2021^25^	?	?	+	?	+	+	+	?	?	+	-	+	+	+	?	+	-	*+*
Chen et al, 2022^26^	?	?	?	-	+	+	?	?	?	?	-	+	+	?	-	?	-	*?*
Chen et al, 2022^27^	?	?	+	?	+	+	?	?	?	+	-	+	+	?	?	?	-	*?*
Cheng et al, 2017^28^	?	?	+	-	+	+	+	?	?	+	-	+	+	+	-	+	-	*+*
Hao et al, 2020^29^	?	?	?	-	+	+	?	?	?	?	-	+	+	?	-	?	-	*?*
Hou et al, 2022^30^	?	?	+	-	+	+	+	?	?	+	-	+	+	+	-	+	-	*+*
Hou et al, 2023^31^	?	?	+	?	+	+	?	?	?	+	-	+	+	?	?	?	-	*?*
Ji et al, 2024^32^	?	?	+	-	+	+	+	?	?	+	?	+	+	+	-	+	?	*+*
Li et al, 2020^33^	?	?	?	?	+	+	?	?	?	?	-	+	+	?	?	?	-	*?*
Li et al, 2021^34^	?	?	+	?	+	+	+	?	?	+	?	+	+	+	?	+	?	*+*
Li et al, 2022^35^	?	?	+	-	+	+	?	?	?	+	-	+	+	?	-	?	-	*?*
Liu et al, 2023^36^	?	?	+	?	+	+	+	?	?	+	-	+	+	+	?	+	-	*+*
Liu et al, 2024^37^	?	?	+	-	+	+	?	?	?	+	-	+	+	?	-	?	-	*?*
Lv et al, 2021^38^	?	?	+	?	+	+	?	?	?	+	-	+	+	?	?	?	-	*?*
Lv et al, 2023^39^	?	?	+	?	+	+	?	?	?	+	-	+	+	?	?	?	-	*?*
Ning et al, 2020^40^	?	?	+	-	+	+	?	?	?	+	-	+	+	?	-	?	-	*?*
Ning et al, 2023^41^	?	?	+	-	+	+	?	?	?	+	-	+	+	?	-	?	-	*?*
Perez-Herrera et al, 2024^42^	?	?	+	?	+	+	?	?	?	+	-	+	+	?	?	?	-	*?*
Qiu et al, 2024^43^	?	?	+	?	+	+	?	?	?	+	-	+	+	?	?	?	-	*?*
Shao et al, 2020^44^	?	?	+	-	+	+	?	?	?	+	-	+	+	?	-	?	-	*?*
Shao et al, 2023^45^	?	?	+	-	+	+	?	?	?	+	-	+	+	?	-	?	-	*?*
Shao et al, 2020^46^	?	?	+	-	+	+	?	?	?	+	-	+	+	?	-	?	-	*?*
Shao et al, 2023^47^	?	?	+	-	+	+	?	?	?	+	-	+	+	?	-	?	-	*?*
Steyaert et al, 2023^48^	?	+	+	-	+	+	+	?	+	+	-	+	+	+	-	+	-	*+*
Subramanian et al, 2021^49^	?	?	+	?	+	+	?	?	?	+	-	+	+	?	?	?	-	*?*
Subramanian et al, 2024^50^	?	?	+	-	+	+	+	?	?	+	-	+	+	+	-	+	-	*+*
Sun et al, 2018^51^	?	?	+	?	+	+	?	?	?	+	-	+	+	?	?	?	-	*?*
Tan et al, 2022^52^	?	?	+	-	+	+	?	?	?	+	-	+	+	?	-	?	-	*?*
Vale-Silva et al, 2021^53^	?	?	+	?	+	+	?	?	?	+	-	+	+	?	?	?	-	*?*
Vollmer et al, 2024^54^	-	?	+	?	+	+	+	-	?	+	-	+	+	+	-	+	-	*+*
Wang et al, 2021^55^	?	?	+	-	+	+	?	?	?	+	-	+	+	?	-	?	-	*?*
Wang et al, 2023^56^	?	?	+	-	+	+	?	?	?	+	-	+	+	?	-	?	-	*?*
Wei et al, 2023^57^	?	?	+	?	+	+	+	?	?	+	-	+	+	+	?	+	-	*+*
Wu et al, 2023^58^	?	?	+	-	+	+	?	?	?	+	-	+	+	?	-	?	-	*?*
Xie et al, 2024^59^	?	?	+	-	+	+	?	?	?	+	-	+	+	?	-	?	-	*?*
Zeng et al, 2020^60^	?	?	+	?	+	+	+	?	?	+	-	+	+	+	?	+	-	*+*
Zeng et al, 2021^61^	?	?	+	-	+	+	+	?	?	+	-	+	+	+	-	+	-	*+*
Zhan et al, 2021^62^	?	?	+	-	+	+	?	?	?	+	-	+	+	?	-	?	-	*?*
Zhang et al, 2020^63^	?	?	+	-	+	+	?	?	?	+	-	+	+	?	-	?	-	*?*
Zhao et al, 2023^64^	?	?	+	-	+	+	?	?	?	+	-	+	+	?	-	?	-	*?*
Zheng et al, 2024^65^	?	?	+	?	+	+	?	?	?	+	-	+	+	?	?	?	-	*?*
Zhou et al, 2023^66^	?	?	+	?	+	+	+	?	?	+	-	+	+	+	?	+	-	*+*
Zhou et al, 2023^67^	?	-	+	?	+	+	+	?	-	+	-	+	+	+	-	+	-	*+*
Zhou et al, 2024^68^	?	?	+	?	+	+	?	?	?	+	-	+	+	?	?	?	-	*?*
Zhu et al, 2023^69^	?	?	+	-	+	+	?	?	?	+	-	+	+	?	-	?	-	*?*

Source: High concern (-, shaded red), Unclear (?, shaded yellow), Low concern (+, shaded green).

#### Participant

All studies had an unclear (n = 47) or high concern (n = 1) of bias in the participant domain. This was predominantly due to a lack of information provided about the TCGA datasets used, including recruitment methods, setting, and inclusion and exclusion criteria, reflecting limitations in reporting at the level of the prediction model studies rather than deficiencies in the underlying source datasets. Only a couple of studies included additional detail, such as recruitment dates^24^ and number of sites.^33^ Where additional datasets were used, a greater level of information was usually provided, but this still provided an incomplete picture of the source of data in all studies.^22,39,42,48,59,65,67^

Individual study criteria for inclusion or exclusion, where stated, were usually data based; defined by presence in a given TCGA data subset, by data quality and/or by completeness of matched data across modalities. Only 2 studies had clinically orientated inclusion criteria.^46,54^ Presentation of participant characteristics was poor, with 18 studies (38%) providing no description of the cohort used, see Table 1. The remaining studies presented variable coverage of participant demographics, disease and treatment information. In 1 study, this was presented comprehensively enough to assess the representativeness of the dataset.^54^ In this study of oral squamous cell carcinoma, participant demographics were broadly representative of the USA population,^70^ however were skewed towards a population with a higher pathologic stage of disease.

#### Predictor

Studies generally provided no information about data acquisition processes making it difficult to assess similarity of predictor assessment. Within the TCGA dataset, there are known to be differences in protocols and processes between contributing institutions which are sufficient to cause batch effect in both sequencing and imaging data.^71-75^ Whilst the variation was not well described, a few of the papers outlined pre-processing steps in one or both modalities designed to address these issues. Only one study described performing these across both modalities and was rated as low concern.^48^

In one of the studies, participants from one contributing site in the TCGA dataset were removed after persistent variation in stain post processing, which raised concern for selection bias.^67^ Another study integrated -omic data from different datasets, generated by different methods without clear discussion about the comparability of these methods and any consequent differences when used to infer the homologous recombination (HRD) status from them.^22^

#### Outcome

Assessments of quality and RoB were generally low due to nature of the outcome (death), which is not so sensitive to bias. However, studies which did not provide enough information to appreciate the intended outcome prediction time horizon or assess the length of follow up of the patients used in the study were rated as unclear.^26,29,33^

#### Analysis

In this domain, there was high concern of quality in 25 studies and of risk of bias in 45 studies. Common factors contributed to these decisions as outlined below.

Assessment of adequacy of sample size in ML and DL approaches is difficult due to the influence of regularisation techniques, shrinkage methods, and hyperparameter tuning strategies aimed to prevent overfitting. The application of the traditional “rule of thumb” 10 events per variable are inappropriate in this setting and there are no clearly defined minimum criteria. As shown by Collins et al. (2021), model complexity and tuning parameters substantially increase data demands, and the number of outcome events may be more influential than total sample size alone.^76^ The use of cross validation, which increases the effective size of the test set thereby reducing over optimism in the results further complicates assessment. Recent guidance encourages authors to justify their sample size for the reader.^77^ However, none of the studies presented any calculation or justification of their sample size and many were missing key data such as sample size (n = 1), number of events (n = 26), number of candidate (n = 24) or final predictors (n = 9) used in the models making it difficult to assess event per variable.

Another key consideration was the common use of a complete case analysis approach, with subjects with missing data modalities excluded and variables with missing values in included patients often removed. This approach can introduce bias if the missingness is not at random. However, it is also appreciated to make sense as part of predictor reduction strategy. Three studies described a proactive approach to data missingness and are discussed in the model section below.

All studies reported measures of discrimination, which test the model’s ability to correctly distinguish between patients who experience the event (eg, death) earlier versus later. However, only 4 evaluated calibration, which refers to how closely a survival prediction model’s estimated probabilities of survival match the observed survival outcomes. These are complementary aspects of model performance and evaluating only 1 can miss serious model flaws—for example, a model may rank patients correctly (good discrimination) but still systematically over- or under-predict risk (poor calibration), leading to biased clinical decisions. The limited reporting of calibration substantially restricts assessment of clinical reliability and undermines the interpretability of reported performance gains, particularly in the context of clinical translation where absolute risk estimates are required for decision-making.

Additionally, the presentation of results did not include metrics of variability in 6 studies and in a further 19 the metric used was unclear.

#### Overall Quality and Risk of Bias

None of the studies had high or unclear ratings in just a single domain, indicating literature in this area faces broad methodological and reporting issues rather than isolated methodological flaws. In particular, incomplete participant description and missing validation detail limit confidence in the reported performance metrics and suggest current results should be treated as exploratory.

#### Overall Applicability

This systematic review was designed to be broad in scope. All studies were assessed as low concern in the participant domain as all were studies of cancer patients. In the predictor domain, all studies were rated as low concern, clearly using high-throughput -omic data and pathology whole slide images in their models. In the outcome domain, all studies were predictive of survival. However, 31 studies were not explicit about an outcome of OS and used ambiguous phrasing such as “cancer survival” or just “survival.” The most used dataset in this study (TCGA) is known to have associated cancer specific and OS data and as such specific phrasing was sought or the applicability of the study was assessed as unclear.^78^

### Data Synthesis Results

#### Datasets

**Figure 3.**
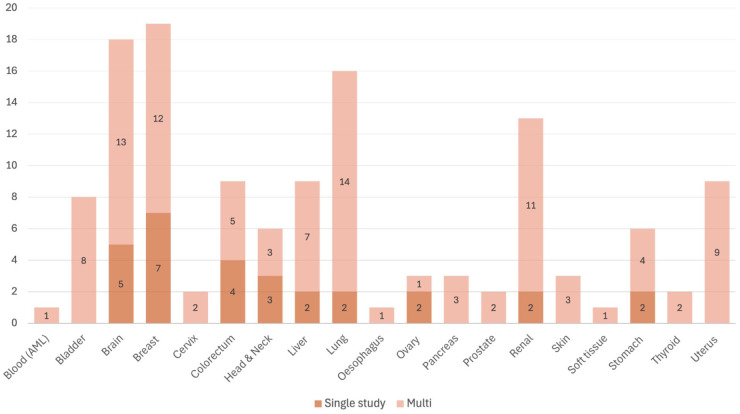
Cancer types represented across the studies in this review. The number of studies (*y*-axis) are presented by organ systems (*x*-axis), showing a differentiation between whether they were part of multi- or single cancer studies. Nineteen studies developed models across multiple cancer subtypes and organs, with the remaining 29 being single cancer type studies. The most commonly studied cancers were those of the brain, breast and lung.

Twenty-nine studies focussed on a cancer of a single organ/anatomical region, while 19 were multi-tissue studies. Nineteen different organs were studied, with the most studied being brain, breast, lung and kidney. Figure 3 summarises frequencies of the cancer domains studied.

Alongside WSIs, gene expression (mRNA) data was used in all studies, with additional -omics used in variable combinations; somatic mutation data (n = 9), micro-RNA (n = 5), copy number variation (CNV; n = 16), single nucleotide variation (SNV; n = 3), DNA methylation (n = 5) and protein expression (n = 5). Clinical data was integrated as an additional modality into 15 models and the variables used most commonly were age, gender and stage. The number of participants used per model of interest ranged from 63 to 11 160.

The TCGA dataset was used in some capacity in all studies, 47 for training, and in 3 studies as an independent external dataset. TCGA is a well-known, multi-institutional public dataset comprising mixed -omic data and WSIs for over 11 000 participants from the USA spanning 33 cancer types.

Other datasets used in multimodal model development and/or validation were from Clinical Proteomic Tumour Analysis Consortium (CPTAC),^48,65^ Memorial Sloan Kettering Cancer Centre,^22^ Molecular Taxonomy of Breast Cancer International Consortium (METABRIC),^42^ NCT-CRC-HE-100,^39^ Paediatric Brain Tumour Atlas,^48^ National Lung Screening Trial,^65^ Tumour infiltrating lymphocytes in breast cancer (TIGER),^42^ Ruijin Hospital (Shanghai)^59^ and Wayne State University.^67^ The countries from which data were used include USA, China, Belgium and Netherlands. The predominant usage of open access public data repositories meant data from these studies was largely assessed as available, however only 18 studies included specific statements relating to data availability – asterisks in Table 1 indicate where data availability was assumed. Those that did not, all exclusively used the TCGA dataset.

Reliance on the TCGA dataset is a concern for this field. Such dependence increases the risk that models are inadvertently learning dataset-specific artefacts rather than generalisable and true biological patterns.

#### Modelling Methods

In this review, the model of interest was defined as the best performing model. Many of the studies, created more than one model by either: independently training and evaluating the same model architecture on datasets of multiple cancer types, combining different -omic combinations with image and/or clinical data into the model, or by testing multiple different model architectures or fusion approaches. Where additional models were present in a study, this is indicated in Supplemental Table S1.

##### Models

Modelling approaches included cox regression models with ML-based regularisation and/or feature selection (n = 4),^28,44-46^ classical ML methods (n = 13) and DL methods (n = 31). ML methods included 7 models based on random forest,^24,25,32,34,54,60,61^ a multi constraint latent representation framework,^41^ 2 variants of canonical correlation analysis,^49,50^ two multiple kernel learning models^51,63^ and a multiview subspace learning model.^64^ Of the 31 DL models, 25 used a cox-based loss as the objective function for survival prediction (log partial or negative log partial likelihood), but cross entropy loss^26,65^ and binary cross entropy loss^37,55^ were also used. One study used a custom likelihood and ranking loss function (DeepHit).^33^

Within the models, fusion of data at a feature level was more common (n = 42) than decision level fusion.^22,31,48^ Three studies used a hybrid approach, processing 1 modality to a decision level (risk score) for integration with features of another modality in the prediction model.^30,32,57^ Studies using feature fusion methods leveraged mechanisms to capture cross-modal interactions, most commonly through attention-based approaches, where models compare different parts of the input, assign relevance scores, and weight them to focus on the most important information (see Supplemental Table S1).

While most models required fully matched datasets, 4 studies implemented explicit strategies to accommodate missing data modalities without excluding patients.^23,31,43,53^ Qiu et al. and Hou et al. used latent-space reconstruction—via variational autoencoders and online masked autoencoders, respectively—to recover missing features.^31,43^ In contrast, Vale-Silva and Rohr used multimodal dropout during training and standard imputation for partial missingness, while Cheerla and Gevaert dynamically reweighted available modalities during fusion.^23,53^ Despite differing mechanisms, all approaches aimed to maintain performance under incomplete input and reported reduced accuracy when modalities were missing. Models lacking such strategies may be vulnerable to overfitting and underperformance in real-world, incomplete datasets.

Model code was described as available in 25 studies, partially available code (as “related core code”) in 1 study^41^ and not commented on in the remaining 22 studies. Using links to code repositories, in 4 studies code could not be found,^28^ was described as “coming soon”^68^ or had been taken down and was now only available on request.^41,64^

##### Data Pre-Processing

These complex data require pre-processing before they can be used in a model and reduction in the dimension of the also data helps to reduce model overfitting. Such steps were variably described in these studies and are summarised below and in Supplemental Table S1.

Most researchers performed tissue segmentation and/or stain normalisation of WSIs before dividing the original WSI into patches, ranging from 16 × 16 to 5000 × 5000 pixels, most commonly 1000 × 1000 pixels (n = 12) or 512 × 512 pixels (n = 11). Four studies used multiscale patches.^27,38,39,56^ Further selection of patches for inclusion was commented on in 32 studies and was performed at random (n = 9), by RGB density (n = 8), nuclear density (n = 2), proximity to RGB mean (n = 1), based on expert defined regions of interest (n = 9), by exclusion of patches with low tissue coverage (n = 2) or for not meeting feature extraction model criteria (n = 1). Image features were generated equally by hand-crafted (n = 23) or learned approaches (n = 23) with 2 studies using a combined approach. CellProfiler was the most popular tool for generating hand-crafted features (n = 17) and ResNet50 the most popular architecture for learned features (n = 7). Some models incorporated graph-based ML,^49,50^ graph neural network,^31,65^ transformer^38,39,56^ or foundation model^69^ approaches for feature generation.

Pre-processing of -omic data, where described, included removal of missing data, normalisation of read counts, and discretisation of expression or CNV data into categorical variables. The 2 studies which described correction for batch effect, both used ComBATSeq.^48,65^ In some studies, -omic data was used directly, however several performed feature generation, including; knowledge-based approaches, such as gene set enrichment analysis (n = 1) and inference of known mutational signatures (n = 2), gene modularisation by data driven methods, including Weighted Gene Co-expression Network Analysis (n = 6) and local maximal Quasi-Clique Merger (n = 4), and generation of feature representations by ML methods (n = 15).

Steps to reduce number of predictors were described in most studies, see Supplemental Table S1, and used a combination of data and knowledge-based approaches. The heterogeneity of approaches to feature generation (predictors) makes it difficult to compare number of predictors across studies. It was also challenging to determine the predictor number in many of the models. Particularly those where features are generated via DL and have cross-modal interactions resulting in complex representations from the WSI and -omic inputs.

#### Model Performance and Evaluation

All studies performed some form of internal validation, with most using a cross-validation approach (n = 44) and the remaining few using random data splitting (n = 3) and bootstrapping (n = 1).

As discussed, studies focussed on measures of discrimination for reporting model performance. The c-index was the most commonly reported metric (39/48). It is a measure of the ability of the model to rank patients in terms of their predicted risk of event by indicating the proportion of all usable patient pairs in which the patient with the shorter observed survival time also has the higher predicted risk. A c-index of 1 indicates perfect performance and 0.5 is equivalent to random predictions. Results across these studies ranged from 0.550 to 0.857. Studies which additionally modelled survival as a binary classification task (ie, predicting longer or shorter survival based on defined thresholds) reported AUC metrics (n = 14). An AUC of 1 reflects perfect classification and 0.5 random guessing. Results across studies ranged 0.662 to 0.932. Time-specific AUC, which measures how well the model can distinguish between classes at a set time point, was reported in 8 studies with results across multiple time points but most commonly for 1-, 3- and 5-year survival. Two studies performed neither of these analyses, instead assessing model performance by hazard ratios^42^ and F1 score.^49^

Five studies performed external validation on their multimodal model,^48,59,64,65,67^ with an additional 4 studies undertaking external validation but only of unimodal models in their study.^24,25,57,61^ Results of multimodal external validation ranged from 0.583 to 0.778 (c-index).

Only 7 studies performed clinical utility analyses, all via decision curve analysis.^24,25,30,32,34,60,61^ Four additionally explored end-user presentation of the model via development of nomograms^24,30,32,34^ and a further study generated an online tool.^57^

##### Unimodal Performance Comparison

**Figure 4.**
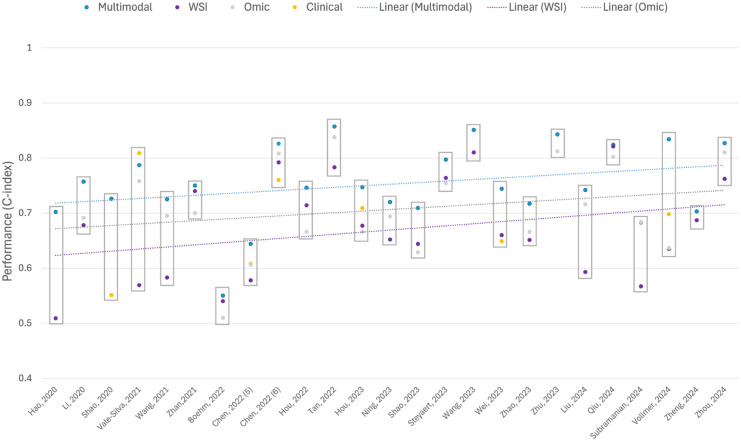
Comparison of multimodal and unimodal model performance using c-index. The 25 studies which reported the c-index for unimodal comparison are shown above. Studies are ordered by year of publication (*x*-axis) with regression lines to show a trend of performance (c-index, *y*-axis) over time. Different studies performed different unimodal comparisons reflected by incomplete data points for several of the studies. Where multiple -omics were evaluated separately, the best performing is charted here. A further 7 studies evaluated unimodal performance against the multimodal model but generated AUC (n = 6) or F1 metrics (n = 1) so were not included in this comparison. Supplemental Table S1, indicates all studies which undertook unimodal performance comparisons.

Thirty-four out of the 48 studies made a performance comparison between the multimodal model of interest and unimodal models based on clinical, -omic or image data. Where the c-index was available as a metric (n = 25), these have been plotted in Figure 4. Studies which made a unimodal comparison but assessed this by another metric (AUC = 10, F1 = 1) could not be included. In all but one study, multimodal models outperformed unimodal models but the extent of improvement in prediction is highly variable. Vale Silva and Rohr’s MultiSurv model showed greater performance on clinical data (c-index 0.809) than with their multimodal model (c-index 0.787, multiomics + WSI), or image (c-index 0.569) and -omic (c-index 0.758, mRNA) based models.^53^ The relatively modest performance gain observed for most multimodal models over unimodal comparators suggest that WSI and -omic data may carry signals with significant overlap rather than distinct contributions. Indeed, morphological patterns associated with underlying mutational features are already well-established for some tumours. The technical variation of methods within these studies may also partly account for the variability of multimodal improvement observed across studies, with potential for significant noise introduction through patch sampling and processing methods or suboptimal alignment and weighting of different data modes through fusion approaches.

##### Other Performance Comparisons

Performance across cancer types was compared where there were results presented in greater than 5 studies, see Supplemental Figure S2. This synthesis used data from all datasets evaluated in the studies for the model of interest, including alternative dataset results from multi-cancer studies. In this descriptive comparison, variation in performance across different cancer types is observed. Higher c-index values observed for glioma and renal papillary cell carcinoma may reflect that, in these tumour types, both morphology and key molecular alterations are independently diagnostic and formally incorporated into WHO classification frameworks.^79,80^ Complementary histological features and underlying biology patterns likely provides a stronger signal for multimodal models to exploit. In contrast, in cancers with more heterogenous genotype-phenotype relationships such as colorectal or breast cancer, the benefit of multimodal fusion may be in inherently limited.

The number of patients in the studies showed a weak trend towards increased performance with increased sample size. The presence of 400 patients or more appeared to be sufficient to achieve optimal results on internal test sets. Studies with fewer participants generally performed the worse (Supplemental Figure S3). There was no meaningful association between performance and increasing event rate, although only 15 studies reported data sufficiently to be included in this analysis (Supplemental Figure S4). These comparisons are illustrative only, due to heterogeneity in methods and risk of bias noted in this review.

## Discussion

Our review highlights the rapid growth of this research domain in the last 5 years. Models have been developed across a wide spectrum of cancers, representing 19 organ systems and 23 specific diagnostic subtypes. Similar to previous works, we find that multimodal integration of WSI and -omic data, generally leads to improved prognosis prediction. Despite methodological progress, these models are far from ready for clinical evaluation.

All included studies were judged to be at high or unclear risk of bias, highlighting significant issues in study design, reporting, and validation. As a result of the significant heterogeneity and potential bias in these models, we are not able to more rigorously assess these models by meta-analysis. However, the range in reported performance is wide (c-index, 0.550-0.857) and the gains over unimodal models, where evaluated, are often small. The generally small increments in c-index imply that integration alone does not guarantee better prognostic discrimination, likely reflecting a combination of biological redundancy between WSI and -omic features, and methodological issues that may mask a true effect. As such, we remain uncertain about the potential clinical value of these models for the future.

These results also raise the prospect that multimodal data integration will not universally add value across the oncology domain, instead adding value in a cancer-specific way dictated by the underlying coupling of tissue morphology and molecular alterations within each cancer. However, the current evidence base is characterised by substantial heterogeneity in modelling approaches, predictor sets, and outcome definitions, which limits the feasibility of robust subgroup analyses within this single broad review. Future benchmarking across tumour types may help clarify where multimodal approaches offer the most meaningful advantage and should be further developed.

ML methods are an attractive prospect, able to flexibly handle high-dimensional, non-linear data. However, despite these perceived advantages many studies of ML prediction models in other domains have found no additional benefit of ML over traditional models.^8^ Given the expense of data generation, that is not part of routine clinical workflows (eg, transcriptomic data), the gain would need to be substantial to be used clinically. Model complexity is another factor in the cost of implementing these multimodal models and only 1 study in this review evaluated compute requirements of their model.^35^ Future studies must compare models against standard clinical prediction methods and begin to evaluate the clinical utility and cost benefit of their models, and not just seek to outperform the discrimination metrics of competing models.

These shortcomings seem reflective of the immaturity of this field in which methodological exploration for handling complex image data and innovation of ML approaches in predictive modelling has been the focus so far.

DL-based models predominate in these studies, as does feature fusion of data. Some of the more recent DL models make use of graph networks, transformers and foundation models – keeping pace with DL developments in other fields. Whilst many studies develop approaches to capture cross-modal interactions and maximise the benefit of this complementary data, relatively fewer have explored models which are robust to missing data modalities – a common real-world scenario. Of concern, most studies lacked clinical contextualisation, with populations often defined by data availability rather than intended use. Important data issues, including assessment of the representativeness of the data for demographic and clinical features, and removal of missing data without consideration and evaluation of whether this is missing at random or not, were almost universally overlooked. The well-known nature of TCGA used by most studies may be leading authors to assume knowledge from readers. This assumption, however, directly limits transparent reporting at the level of the prediction model studies themselves, constraining clear assessment of participant and predictor domains within risk-of-bias frameworks, regardless of the quality or documentation of the underlying source datasets. Yet, there are well-documented batch effects and participant shifts versus the general population for age, race and stage in this dataset, and more detail is important to the interpretation of these models.^71-74,81-83^ Despite reliance on public datasets, open science practices were inconsistently adopted, with limited code and data availability described.

The heavy dependence on the TCGA dataset, used in all studies, may be an indicator that we don’t yet have the necessary data to build and test these models. The whole field may be at risk of overfitting to the features of a single source and creating an illusion of reproducibility. The oncology community need to build more easily accessible datasets with detailed meta data and appropriate longitudinal follow up to provide clear and independent data sets on which to validate models and establish their capacity to generalise beyond TCGA.

### Limitations of the Review

The review protocol was designed to limit bias and maximise inclusion of eligible studies. While controlled vocabulary terms are available for several of the search concepts, the strategy relied primarily on free-text searching. Despite being supplemented by extensive citation searching, it is possible that some relevant studies were not retrieved. The review was restricted to studies published in English and as such may have missed studies in other languages. All stages of screening and data extraction were performed by 2 independent researchers, except for the initial duplicate screening which was performed by a single researcher (with the aid of *Rayyan* software^16^), raising the possibility of incorrectly excluding studies in error.

The significant heterogeneity between the studies in this review precluded more detailed data synthesis and meta-analysis which limited the conclusions that can be made. Due to the rapid expansion of this research area, a future a review focussed on a specific cancer types or specific modelling methods may provide the opportunity for a more rigorous analysis.

Finally, this review focussed on models which directly integrated pathology and -omic data, overlooking studies outside this domain. Most relevant for future consideration may be the evaluation of models which additionally incorporate radiology data,^84,85^ replicating the current clinical paradigm, and models which used -omic and image data in a pipeline. Such approaches generally first generate an -omic prediction from the image, then generate an image-based prediction model.^86,87^ The rising availability and use of spatial -omic data may propel this field forward, bridging the gap between morphology and bulk -omic data for greater biological and interpretable insights.^88,89^

### Current Limitations and Future Recommendations

Our review finds that many aspects of methodology and reporting were suboptimal in these studies, in keeping with findings of other systematic reviews of prognostic models in the oncology domain.^2,8,90^ Whilst there is rapid proliferation of prognostic models in cancer, the translation rate from academic generation to clinical implementation is very low.^8^ The next phase of model development in this domain will benefit from reflections on lessons learnt about how to design clinically useful and usable models.^73^ Future research in this area should adhere to reporting guidance, recently updated to better reflect complexities of ML-based modelling. TRIPOD + AI is a 27-item checklist designed to promote transparent reporting of studies developing, validating or updating prediction models.^91^ The PROBAST + AI tool used in this review also outlines best practice approaches to data selection and handling, modelling, and reporting.^20^ Together, these frameworks highlight that methodological quality and transparent reporting are tightly linked and both are necessary for meaningful evaluation and clinical translation of prognostic models. Without better reporting, apparent progress in multimodal cancer prognosis prediction may be overstated.

Even for well-developed and validated models, clinical implementation is not realistic in the near future. Multimodal models are being developed in advance of the widespread clinical availability of high-throughput -omic and WSI data. In the UK, for example, approximately 10% of the molecular workload in the National Health Service is whole genome sequencing with the majority of the work centred on targeted mutation testing and smaller gene panels – there is no routine RNA sequencing.^92^ In parallel, digitisation of pathology services across the UK is not yet complete.^93^ Understanding of where and when these models may provide additional benefit to patients from well-conducted research may inform how such services develop.

Finally, research in this field will not progress without wider use and availability of additional datasets. So far, generation of these complex data has largely been the reserve of publicly funded initiatives. Other pan cancer projects such as the Applied Proteogenomic Organisational Learning Outcomes (APOLLO) network^94^ and the Cancer Moonshot Biobank,^95^ are increasing the availability of publicly accessible -omic data with matched pathology and radiological imaging data. Genomics England’s 100 000 Genomes Project Multimodal Programme is a large apply to access dataset also on the horizon.^96^ However, existing “apply to access” datasets, such as the Children’s Brain Tumour Tissue Consortium^97^ and METABRIC (breast)^98^ seem under used in this field so far. The reason for this is unclear and may be multifactorial. Critical to clinical implementation will be increased availability and use of datasets from more diverse populations (genetic ancestry, geographic, environmental) and datasets which are representative of both the medical diversity of a condition, and the technical diversity of the data generated.^99^

## Conclusion

This review of machine learning-based multimodal predictive models found 48 eligible studies predicting overall survival in cancer participants. This demonstrates significant growth in this research field since first works were identified in 2017. Progress has centred on methodological innovation, particularly through deep learning approaches. However, studies are limited by poor reporting, limited validation and lack of clinical contextualisation. To advance towards clinical impact, future research must prioritise transparent reporting, large diverse datasets, meaningful comparison to clinical standards, and a clear demonstration of how and where these models might be used in practice. Striking the right balance between model complexity, computational demands, and meaningful gains in predictive performance will be essential for real-world implementation—particularly in resource-constrained healthcare settings where cost-effectiveness is critical.

## Supplemental Material

sj-pdf-1-cix-10.1177_11769351261434523 – Supplemental material for AI in Cancer Prognosis: A Systematic Review of Multimodal Models Combining Pathology Images and High-Throughput Omics Supplemental material, sj-pdf-1-cix-10.1177_11769351261434523 for AI in Cancer Prognosis: A Systematic Review of Multimodal Models Combining Pathology Images and High-Throughput Omics by Charlotte Jennings, Andrew Broad, Lucy Godson, Emily Clarke, David Westhead and Darren Treanor in Cancer Informatics
