# Supplementary material for: AI in Cancer Prognosis: A Systematic Review of Multimodal Models Combining Pathology Images and High-Throughput Omics
Source: Cancer Inform. 2026 May 11;25:11769351261434523. doi: 10.1177/11769351261434523 (PMC13172687; doi:10.1177/11769351261434523)
Supplement: sj-pdf-1-cix-10.1177_11769351261434523 – Supplemental material for AI in Cancer Prognosis: A Systematic Review of Multimodal Models Combining Pathology Images and High-Throughput Omics [file sj-pdf-1-cix-10.1177_11769351261434523.pdf]

Supplementary materials

Literature search strategy........2
1.1 Screening algorithms........7
1.1.1 Abstract screening........7
1.1.2 Full text screening........8
1.2 Data extraction template summary........9
1.3 Author definitions........10
1.3.1 Model Categories used in the review........10
1.3.2 Fusion approach definitions........11
1.4 PRISMA 2020 checklists........11
1.4.1 PRISMA 2020 Main Checklist........11
1.4.2 PRISMA Abstract Checklist........18
1.4.3 SWiM 2021 checklist........20
1.5 Additional results........23
1.5.1 Figure S1. Modelling method trends over time........26
1.5.2 Figure S2. Model performance by cancer dataset........27
1.5.3 Figure S3. Model performance by sample size........28
1.5.4 Figure S4. Model performance by event rate........28

Literature search strategy

Search strategies for the three databases shown below with numbers of results for each indicated.

1. PUBMED (12/08/2024)

Search	Term [title/abstract]	Number
Pathology whole slide images		
#1	Pathology	427135
#2	Histo*	2238976
#3	“H&e”	19770
#4	“Haematoxylin and eosin”	3688
#5	Imag*	751150
#6	“digital pathology”	2714
#7	“whole slide image*”	2192
#8	“WSIs”	863
#9	((#1 OR #2 OR #3 OR #4) AND #5) OR # 6 OR #7 OR #8	89536
Omic data		
#10	Genom*	874583
#11	Omic*	44061
#12	Proteom*	146305
#13	Epigen*	135526
#14	Transcriptom*	166893
#15	Molecular	1828450
#16	#10 OR #11 OR #12 OR #13 OR #14 OR #15	27992170
Pathogenomic		
#17	Pathogenom*	268
#18	(#9 AND #16) OR #17	4932
Machine learning method		
#19	“deep learning”	68880
#20	“machine learning”	121049

#21	"neural network"	80989
#22	"artificial intelligence"	55905
#23	Fusion	255302
#24	Integrat*	792949
#25	Multimodal	67428
#26	multi-modal	7934
#27	(#19 OR #20 OR #21 OR #22) AND (#23 OR #24 OR #25 OR #26)	39060
Cancer		
#28	Cancer*	245687
#29	Tumo?r*	2157883
#30	Carcinoma*	815576
#31	Sarcoma*	118675
#32	Onco*	449173
#33	#28 Or #29 OR #30 OR #31 OR #32	4062912
Final search		
#33	#18 AND #27 AND #33	215
#33	English, human	128

2. OVID: Embase classic+ Embase, MEDLINE (R) ALL (12/08/2024)

**Search**	**Term [title/abstract]**	**Number**
Pathology whole slide images		
#1	Pathology	964013
#2	Histo*	5508575
#3	"H&e"	718077
#4	"Haematoxylin and eosin"	13620
#5	Imag*	1753707
#6	"digital pathology"	5003
#7	"whole slide image*"	5487
#8	"WSIs"	2156
#9	((#1 OR #2 OR #3 OR #4) AND #5) OR # 6 OR #7 OR #8	247780

Omic data

#10	Genom*	1902327
#11	Omic*	92389
#12	Proteom*	322280
#13	Epigen*	300859
#14	Transcriptom*	365122
#15	Molecular	3944775
#16	#10 OR #11 OR #12 OR #13 OR #14 OR #15	6054934

Pathogenomic

#17	Pathogenom*	501
#18	(#9 AND #16) OR #17	14278

Machine learning method

#19	"deep learning"	128669
#20	"machine learning"	231297
#21	"neural network"	163613
#22	"artificial intelligence"	102044
#23	Fusion	567992
#24	Integrat*	17333754
#25	Multimodal	138719
#26	multi-modal	19255
#27	(#19 OR #20 OR #21 OR #22) AND (#23 OR #24 OR #25 OR #26)	76067

Cancer

#28	Cancer*	5827011
#29	Tumo?r*	5190000
#30	Carcinoma*	1942422
#31	Sarcoma*	274519
#32	Onco*	1121283
#33	#28 Or #29 OR #30 OR #31 OR #32	9712811

Final search

#33	#18 AND #27 AND #33	528
#33	Limit [English language]	522
#34	Limit [humans]	433
#35	Remove duplicates from #34	320

3. CENTRAL

**Search**	**Term [title/abstract/keyword]**	**Number**
Pathology whole slide images		
#1	Pathology	83423
#2	Histo*	150560
#3	“H&E”	701
#4	“Haematoxylin and eosin”	386
#5	Imag*	40806
#6	“digital pathology”	82
#7	Whole NEXT slide NEXT image*	73
#8	“WSIs”	16
#9	((#1 OR #2 OR #3 OR #4) AND #5) OR # 6 OR #7 OR #8	9659
Omic Data		
#10	Genom*	9172
#11	Omic*	927
#12	Proteom*	2021
#13	Epigen*	1846
#14	Transcriptom*	2042
#15	Molecular	30428
#16	#10 OR #11 OR #12 OR #13 OR #14 OR #15	42243
Pathogenomic		
#17	Pathogenom*	1
#18	(#9 AND #16) OR #17	515
Machine learning method		

#19	“deep learning”	1207
#20	“machine learning”	3108
#21	“neural network”	1525
#22	“artificial intelligence”	2388
#23	Fusion	10218
#24	Integrat*	48667
#25	Multimodal	9740
#26	multi-modal	1189
#27	(#19 OR #20 OR #21 OR #22) AND (#23 OR #24 OR #25 OR #26)	784
Cancer		
#28	Cancer*	238625
#29	Tumo?r*	100558
#30	Carcinoma*	54398
#31	Sarcoma*	3502
#32	Onco*	104041
#33	#28 Or #29 OR #30 OR #31 OR #32	291641
Final search		
#33	#18 AND #27 AND #33	15
#33	In trials	9

1.1 Screening algorithms

1.1.1 Abstract screening

Signalling questions for inclusion and exclusion of studies in abstract screening		
**#**	**Questions**	**Response & Action**
1	Is this study written in English?	No = Reject "not English"<br>Yes = Next question
2	Is this article a primary research paper?<br>(ie. not a meta-analysis, review, comment, conference abstract, book chapter etc)	No = Reject "wrong publication type"<br>Yes = Next question
3	Is this article examining cancer?<br>(ie not benign tumor, dysplasia or other non-cancerous medical conditions)	No = Reject "not cancer"<br>Yes = Next question
4	Are the participants human?<br>(ie. Not cell culture or animal study)	No = Reject "not human"<br>Yes = Next question
5	Is the study concerned with a novel prognostic/prediction model?<br>(ie.not a model developed to predict an existing prognostic factor eg. BRAF status from the images, or only identifying new prognostic factors)	No = Reject "not prognostic"<br>Yes = Next question
6	Is the prognostic outcome overall survival or cancer-specific survival?<br>(exclude disease free progression, risk of recurrence, treatment response predictions)	No = reject "wrong outcome"<br>Yes = next question
7	Was the model developed through machine learning methods?<br>(ie. Not other standard statistical methods)<br>Note: logistic regression accepted as machine learning IF described as such by authors.	No = Reject "not ML"<br>Yes = Next question
8	Are whole slide images of surgical pathology part of the model input?<br>IHC and special stains included, TMAs<br>(Not other imaging modality eg. other pathology imaging technologies such as multiplex IHC or immunofluorescence images, radiological imaging, endoscopy etc AND Not cytology, autopsy, toxicology, forensics)	No = Reject "no WSIs"<br>Yes = Next question
9	Is high throughput molecular/omic data part of the model input?<br>(ie.genomes, transcriptomes, proteomes, epigenetic data but NOT simple point mutation testing outcomes eg. BRAFV600E wt/mutant).<br>Note: Spatial transcriptomic data using multiplex imaging accepted where light microscope WSI's also included but not as the only image modality.	No = Reject "no omics"<br>Yes = Next question

1.1.2 Full text screening

Signalling questions for inclusion and exclusion of studies in full-text screening		
**#**	**Question**	**Response & Action**
1	Is this study written in English?	No = Reject "not English"<br>Yes = Next question
2	Is this article a primary research paper?<br>(ie. not a meta-analysis, review, comment, conference abstract, book chapter etc)	No = Reject "wrong publication type"<br>Yes = Next question
3	Is this article peer reviewed?<br>Ie. Not pre-print or other type of non-peer reviewed article	No = reject "wrong publication type"<br>Yes = Next question
4	Is this article examining cancer?<br>(ie not benign tumor, dysplasia or other non-cancerous medical conditions)	No = Reject "not cancer"<br>Yes = Next question
5	Are the participants human?<br>(ie. Not cell culture or animal study)	No = Reject "not human"<br>Yes = Next question
6	Is the study concerned with a novel prognostic/prediction model?<br>(ie.not a model developed to predict an existing prognostic factor eg. BRAF status from the images, or only identifying new prognostic factors)	No = Reject "not prognostic"<br>Yes = Next question
7	Is the prognostic outcome overall survival or cancer-specific survival?<br>(exclude disease free progression, risk of recurrence, treatment response predictions)	No = reject "wrong outcome"<br>Yes = next question
8	Was the model developed through machine learning methods?<br>(ie. Not other standard statistical methods)<br>Note: logistic regression accepted as machine learning IF described as such by authors.	No = Reject "not ML"<br>Yes = Next question
9	Are whole slide images of surgical pathology part of the model input?<br>IHC and special stains included, TMAs<br>(Not other imaging modality eg. other pathology imaging technologies such as multiplex IHC or immunofluorescence images, radiological imaging, endoscopy etc AND Not cytology, autopsy, toxicology, forensics)	No = Reject "no WSIs"<br>Yes = Next question
10	Is high throughput molecular/omic data part of the model input?<br>(ie.genomes, transcriptomes, proteomes, epigenetic data but NOT simple point mutation testing outcomes eg. BRAFV600E wt/mutant).<br>Note: Spatial transcriptomic data using multiplex imaging accepted where light microscope WSI's also included but not as the only image modality.	No = Reject "no omics"<br>Yes = Next question

11	Are these data modalities integrated into a single prognostic model?	No = Reject "not integrated"<br>Yes = include

1.2 Data extraction template summary

The table below summarises the data extraction fields for the systematic review.

The template used was adapted from Fernandex-Felix et al, 2023.

Fernandez-Felix, B.M., López-Alcalde, J., Roqué, M. *et al.* CHARMS and PROBAST at your fingertips: a template for data extraction and risk of bias assessment in systematic reviews of predictive models. *BMC Med Res Methodol* **23**, 44 (2023). https://doi.org/10.1186/s12874-023-01849-0

Adaptations included updating for PROBAST+AI and to enable additional domain specific fields, fields relating specifically to multimodal fusion methods and open science reporting fields for data and code (highlighted in bold). Please see the original publication to access the template.

**Summary info**	
Author; Lead author country; publication year; study title; journal; model name; cancer domain (organ); cancer subtype	
**Study info**	
Source of data	Source of data (retrospective, prospective etc); name of dataset; country of origin; **WSI details (fixation, stain, resolution, scanning platform); omic data types (RNA, miRNA etc)**
Participants	Recruitment method; recruitment dates; study setting; study sites (regions); study sites (no. centres); inclusion criteria; exclusion criteria; participant description; Numbers (participants, **WSI, genes**); participant characteristics (**demographic information available (list); disease information available (list); treatment info provided (list)**)
Outcome	Outcome; outcome definition; type of outcome (single, combined); assessed without knowledge predictors (y/n);

	predictors part of outcome (y/n); time of outcome occurrence
Candidate predictors	Number assessed; type; timing of measurement; predictor measurement similar for all (y/n); predictors blinded for outcome (y/n); handling continuous predictors.
Sample size	Number participants; number events; epv/epp
Missing data	Number participants with missing data; handling of missing data
Model development	Modelling method; **method for generating WSI predictors (pre-processing, sampling resolution, patch size, patch number, patch selection, image feature generation, no. features);** selection WSI predictors; **omic predictor generation (pre-processing, handling missing values, gene feature generation, no. gene features);** selection of omic predictors. **Method multimodal fusion (feature, decision, combination**); method selection during modelling' methods to prevent overfitting
Model performance	Calibration; discrimination; overall; clinical utility
Model evaluation	Internal validation; external validation; model comparison (unimodal, alt multimodal)
Results	Final no predictors in model; alternative presentation of model
Interpretation	Explainability/feature importance methods
Observations	**Data availability; code availability; funding**

1.3 Author definitions

1.3.1 Model Categories used in the review

1. **Regularised Regression Models (e.g., LASSO-Cox, Elastic Net)**

Definition: Models using Cox or logistic regression with machine learning-based regularisation to improve generalisation and automate feature selection.

2. **Classical Machine Learning Models (non-DL)**

Definition: Random forests, support vector machines, gradient boosting, k-nearest neighbors, etc., using structured/hand-crafted features.

3. **Deep Learning Models**

Definition: CNNs, autoencoders, graph networks, transformer and foundation model based approaches

1.3.2 Fusion approach definitions

Feature fusion: Combining features from separate data modalities into a shared representation to generate model predictions.

Decision Fusion: Predictions or decisions from separate unimodal models are combined at a late stage, typically after each modality has been fully processed and independently classified or regressed, to produce a final output.

Hybrid: Combines elements of feature and decision fusion, such as integrating the decision-level output (e.g., risk score) from one modality with the feature-level representation of another.

1.4 PRISMA 2020 checklists

Checklists completed and generated using https://prisma.shinyapps.io/checklist/

1.4.1 PRISMA 2020 Main Checklist

Topic	No.	Item	Location where item is reported
**TITLE**			
**Title**	1	Identify the report as a systematic review.	Page 1, title
**ABSTRACT**			
**Abstract**	2	See the PRISMA 2020 for Abstracts checklist	
**INTRODUCTION**			

Topic	No.	Item	Location where item is reported
**Rationale**	3	Describe the rationale for the review in the context of existing knowledge.	Introduction, page 3-5
**Objectives**	4	Provide an explicit statement of the objective(s) or question(s) the review addresses.	Introduction, page 5
**METHODS**			
**Eligibility criteria**	5	Specify the inclusion and exclusion criteria for the review and how studies were grouped for the syntheses.	Methods; eligibility criteria, page 6
**Information sources**	6	Specify all databases, registers, websites, organisations, reference lists and other sources searched or consulted to identify studies. Specify the date when each source was last searched or consulted.	Methods; data sources and search strategy, page 6
**Search strategy**	7	Present the full search strategies for all databases, registers and websites, including any filters and limits used.	Supplementary file; Literature search strategy, page 1-6
**Selection process**	8	Specify the methods used to decide whether a study met the inclusion criteria of the review, including how many reviewers screened each record and each report retrieved, whether they worked independently, and if applicable, details of automation tools used in the process.	Methods; study selection, page 6-7 & Supplementary file; screening algorithms, page 6-9

Topic	No.	Item	Location where item is reported
**Data collection process**	9	Specify the methods used to collect data from reports, including how many reviewers collected data from each report, whether they worked independently, any processes for obtaining or confirming data from study investigators, and if applicable, details of automation tools used in the process.	Methods; data extraction, page 7-8
**Data items**	10a	List and define all outcomes for which data were sought. Specify whether all results that were compatible with each outcome domain in each study were sought (e.g. for all measures, time points, analyses), and if not, the methods used to decide which results to collect.	Methods; data extraction, page 7 & Supplementary file; data extraction template summary, page 8-9
	10b	List and define all other variables for which data were sought (e.g. participant and intervention characteristics, funding sources). Describe any assumptions made about any missing or unclear information.	Supplementary file; data extraction summary, page 8-9
**Study risk of bias assessment**	11	Specify the methods used to assess risk of bias in the included studies, including details of the tool(s) used, how many reviewers assessed each study and whether they worked independently, and if applicable, details of automation tools used in the process.	Methods; risk of bias assessment, page 8-9

Topic	No.	Item	Location where item is reported
**Effect measures**	12	Specify for each outcome the effect measure(s) (e.g. risk ratio, mean difference) used in the synthesis or presentation of results.	Methods; data synthesis, page 9
**Synthesis methods**	13a	Describe the processes used to decide which studies were eligible for each synthesis (e.g. tabulating the study intervention characteristics and comparing against the planned groups for each synthesis (item 5)).	Methods; data synthesis, page 9
	13b	Describe any methods required to prepare the data for presentation or synthesis, such as handling of missing summary statistics, or data conversions.	N/A
	13c	Describe any methods used to tabulate or visually display results of individual studies and syntheses.	Methods; data synthesis, page 9
	13d	Describe any methods used to synthesize results and provide a rationale for the choice(s). If meta-analysis was performed, describe the model(s), method(s) to identify the presence and extent of statistical heterogeneity, and software package(s) used.	Methods; data synthesis, page 9
	13e	Describe any methods used to explore possible causes of heterogeneity among study results (e.g. subgroup analysis, meta-regression).	N/A

Topic	No.	Item	Location where item is reported
	13f	Describe any sensitivity analyses conducted to assess robustness of the synthesized results.	N/A
**Reporting bias assessment**	14	Describe any methods used to assess risk of bias due to missing results in a synthesis (arising from reporting biases).	N/A
**Certainty assessment**	15	Describe any methods used to assess certainty (or confidence) in the body of evidence for an outcome.	N/A
**RESULTS**			
**Study selection**	16a	Describe the results of the search and selection process, from the number of records identified in the search to the number of studies included in the review, ideally using a flow diagram.	Results, page 9-10 & Figure 1, prism flow diagram
	16b	Cite studies that might appear to meet the inclusion criteria, but which were excluded, and explain why they were excluded.	Results, page 10
**Study characteristics**	17	Cite each included study and present its characteristics.	Table 1
**Risk of bias in studies**	18	Present assessments of risk of bias for each included study.	Table 3

Topic	No.	Item	Location where item is reported
**Results of individual studies**	19	For all outcomes, present, for each study: (a) summary statistics for each group (where appropriate) and (b) an effect estimate and its precision (e.g. confidence/credible interval), ideally using structured tables or plots.	Table 2
**Results of syntheses**	20a	For each synthesis, briefly summarise the characteristics and risk of bias among contributing studies.	N/A
	20b	Present results of all statistical syntheses conducted. If meta-analysis was done, present for each the summary estimate and its precision (e.g. confidence/credible interval) and measures of statistical heterogeneity. If comparing groups, describe the direction of the effect.	N/A
	20c	Present results of all investigations of possible causes of heterogeneity among study results.	N/A
	20d	Present results of all sensitivity analyses conducted to assess the robustness of the synthesized results.	N/A
**Reporting biases**	21	Present assessments of risk of bias due to missing results (arising from reporting biases) for each synthesis assessed.	N/A
**Certainty of evidence**	22	Present assessments of certainty (or confidence) in the body of evidence for each outcome assessed.	N/A

Topic	No.	Item	Location where item is reported
**DISCUSSION**			
**Discussion**	23a	Provide a general interpretation of the results in the context of other evidence.	Discussion, page 17-19
	23b	Discuss any limitations of the evidence included in the review.	Discussion, page 17-19
	23c	Discuss any limitations of the review processes used.	Discussion; limitations of the review, page 19-20
	23d	Discuss implications of the results for practice, policy, and future research.	Discussion, current limitations and future recommendations, page 20-21
**OTHER INFORMATION**			
**Registration and protocol**	24a	Provide registration information for the review, including register name and registration number, or state that the review was not registered.	Methods, page 5
	24b	Indicate where the review protocol can be accessed, or state that a protocol was not prepared.	Methods, page 5
	24c	Describe and explain any amendments to information provided at registration or in the protocol.	N/A
**Support**	25	Describe sources of financial or non-financial support for the review, and the role of the funders or sponsors in the review.	Funding, page 21

Topic	No.	Item	Location where item is reported
**Competing interests**	26	Declare any competing interests of review authors.	Competing interests statement, page 22
**Availability of data, code and other materials**	27	Report which of the following are publicly available and where they can be found: template data collection forms; data extracted from included studies; data used for all analyses; analytic code; any other materials used in the review.	Data availability statement, page 22

1.4.2 PRISMA Abstract Checklist

Topic	No.	Item	Reported?
**TITLE**			
**Title**	1	Identify the report as a systematic review.	Yes
**BACKGROUND**			
**Objectives**	2	Provide an explicit statement of the main objective(s) or question(s) the review addresses.	Yes
**METHODS**			
**Eligibility criteria**	3	Specify the inclusion and exclusion criteria for the review.	Yes
**Information sources**	4	Specify the information sources (e.g. databases, registers) used to identify studies and the date when each was last searched.	Yes
**Risk of bias**	5	Specify the methods used to assess risk of bias in the included studies.	Yes
**Synthesis of results**	6	Specify the methods used to present and synthesize results.	Yes
**RESULTS**			

Topic	No.	Item	Reported?
**Included studies**	7	Give the total number of included studies and participants and summarise relevant characteristics of studies.	Yes
**Synthesis of results**	8	Present results for main outcomes, preferably indicating the number of included studies and participants for each. If meta-analysis was done, report the summary estimate and confidence/credible interval. If comparing groups, indicate the direction of the effect (i.e. which group is favoured).	Yes
**DISCUSSION**			
**Limitations of evidence**	9	Provide a brief summary of the limitations of the evidence included in the review (e.g. study risk of bias, inconsistency and imprecision).	Yes
**Interpretation**	10	Provide a general interpretation of the results and important implications.	Yes
**OTHER**			
**Funding**	11	Specify the primary source of funding for the review.	Yes
**Registration**	12	Provide the register name and registration number.	Yes

From: Page MJ, McKenzie JE, Bossuyt PM, Boutron I, Hoffmann TC, Mulrow CD, et al. The PRISMA 2020 statement: an updated guideline for reporting systematic reviews. MetaArXiv. 2020, September 14. DOI: 10.31222/osf.io/v7gm2. For more information, visit: www.prisma-statement.org

1.4.3 SWiM 2021 checklist

Meta-analysis explanation and elaboration article is: Campbell M, McKenzie JE, Sowden A, Katikireddi SV, Brennan SE, Ellis S, Hartmann-Boyce J, Ryan R, Shepperd S, Thomas J, Welch V, Thomson H. Synthesis without meta-analysis (SWiM) in systematic reviews: reporting guideline BMJ 2020;368:l6890 http://dx.doi.org/10.1136/bmj.l6890

SWiM is intended to complement and be used as an extension to PRISMA			
**SWiM reporting item**	**Item description**	**Page in manuscript where item is reported**	**Other***
Methods			
**1** Grouping studies for synthesis	1a) Provide a description of, and rationale for, the groups used in the synthesis (e.g., groupings of populations, interventions, outcomes, study design)	Page 9, "Data synthesis"	
	1b) Detail and provide rationale for any changes made subsequent to the protocol in the groups used in the synthesis	N/A	
**2** Describe the standardised metric and transformation methods used	Describe the standardised metric for each outcome. Explain why the metric(s) was chosen, and describe any methods used to transform the intervention effects, as reported in the study, to the standardised metric, citing any methodological guidance consulted	Page 9, "Data synthesis:	
**3** Describe the synthesis methods	Describe and justify the methods used to synthesise the effects for each outcome when it was not possible to undertake a meta-analysis of effect estimates	Page 9, "Data synthesis"	
**4** Criteria used to prioritise results for	Where applicable, provide the criteria used, with supporting justification, to select the particular studies, or a particular study, for the main synthesis or to draw conclusions from the synthesis (e.g., based on study design, risk of bias assessments, directness in relation to the review question)	Page 9, "Data synthesis"	

summary and synthesis			
**SWiM reporting item**	**Item description**	**Page in manuscript where item is reported**	**Other***
**5** Investigation of heterogeneity in reported effects	State the method(s) used to examine heterogeneity in reported effects when it was not possible to undertake a meta-analysis of effect estimates and its extensions to investigate heterogeneity	N/A	
**6** Certainty of evidence	Describe the methods used to assess certainty of the synthesis findings	N/A	
**7** Data presentation methods	Describe the graphical and tabular methods used to present the effects (e.g., tables, forest plots, harvest plots).<br>Specify key study characteristics (e.g., study design, risk of bias) used to order the studies, in the text and any tables or graphs, clearly referencing the studies included	Page 9, "Data synthesis"	
Results			
**8** Reporting results	For each comparison and outcome, provide a description of the synthesised findings, and the certainty of the findings. Describe the result in language that is consistent with the question the synthesis addresses, and indicate which studies contribute to the synthesis	Page 9-17, "Results"	
Discussion			
**9** Limitations of the synthesis	Report the limitations of the synthesis methods used and/or the groupings used in the synthesis, and how these affect the conclusions that can be drawn in relation to the original review question	Page 17, performance comparisons. Supplementary Figures 1-4. Page 18,	

		“Limitations of the review”	

PRISMA=Preferred Reporting Items for Systematic Reviews and Meta-Analyses.
*If the information is not provided in the systematic review, give details of where this information is available (e.g., protocol, other published papers (provide citation details), or website (provide the URL)).

1.5 Additional results

Table S1. Supplementary study and data processing details of the models of interest

Author, Year (ref)	Model name (Key dataset)	WSI Pre-processing	Sampling resolution	Patch size	Patch selection	Patches per patient	WSI feature extraction	WSI feature extraction	Selection WSI fetaures	Omic Pre-processing	Omic features	Selection omic features	Intermodality interactions captured by method	Internal validation			External results		Alternative modelling in study:			Comparisons:	
							Approach	Method			Named Method			Approach	Data split	No. folds	Result (metric)	Variability (metric)	Model approach	Data combo	Cancer dataset	Compare unimodal	Alt datset results
Boehm, 2022 (22)	**GHC***, (Ovary) HGSOC	Macenko Stain normalisation	20x*	128x128	-	-	Combination	Tissue classification (ResNet18) and nuclear detection maps	Univariate CPH	Removal of cases with ambiguous HRD status	HRD status inferred from key genes associated	Univariate CPH	No	Random split data	85:15	N/A	-	-	-	Yes	-	Yes	-
Cheerla, 2019 (23)	**Multimodal (Clin+miRNA+mRNA+WSI)**, (Multi - 20) Pancancer	Pre-processed, batch corrected	-	224x224	Select top 20% closest to RGB mean	40	Learned	SqueezeNet CNN	-	-	Deep Highway Networks	All	Via unsupervised similarity loss, and multimodal dropout during training	Random split data	85:15	N/A	-	-	-	Yes	Yes	-	Yes
Chen, 2021 (24)	**HTRS**, (Head & Neck) SCC	Correct illumination	40x	1000x1000	-	20	Hand crafted	CellProfiler	Overlap of SVM-RFE and LASSO-COX selection	-	WGCNA	Genes associated with image features	Partial ly -WGCNA identifies gene modules correlated with image features but no joint learned latent space	Cross-validation	70:30	5	-	-	-	-	-	Yes	-
Chen, 2021 (25)	**Multiomics model** , (Lung) Adenocarcinoma	Correct illumination	40x*	1000x1000	Random	60	Hand crafted	CellProfiler	All	-	-	All proteomic; top 100 somatic mutations, top 100 DEG	No	Cross-validation	50:50	5	-	-	-	Yes	-	Yes	-
Chen, 2022 (26)	**MMF**, (Multi - 14) Pancancer	CLAM tissue segmentation	20x	256x256	From identified tissue regions (unknown number)	-	Learned	ResNet50 (ImageNet)	All	Filter for Genes with >10% CNV or 5% mutation frequency, RNASeq-gene sets	-	-	Shared embedding with attention	Cross-validation	80:20	5	-	-	-	-	Yes	Yes	Yes
Chen, 2022 (27)	**Pathomic Fusion**, (Multi - 2) Glioma	Sparse stain normalisation	20x	512x512, 1024x1024	-	-	Combination	CNN VGG19 (ImageNet), KNN-Cell graph (concatenation of manual, contrastive predictive coding features)	-	-	SNN	-	Gating-based attention + Kronecker product	Cross-validation	80:20	15 (Monte carlo)	-	-	-	Yes	Yes	Yes	Yes
Cheng, 2017 (28)	**Lasso-Cox**, (Multi - 2) CCRCC, Glioma	Unsupervised hierarchical multilevel thresholding (nucleus segmentation)	-	-	-	-	Hand crafted	Unclear	-	Normalised to reads per kilobase per million	lmQCM	-	No	Cross-validation	-	10	-	-	-	-	Yes	-	Yes
Hao, 2020 (29)	**PAGE-NET**, (Brain) Glioblastoma	-	-	256x256	Random	1000	Learned	Pre-trained CNN	All	-	CoxPASNET	KEGG and Reactome datases to filter with biological knowledge	Attention-based fusion	Cross-validation	90:10	-	-	-	-	Yes	-	Yes	-
Hou, 2022 (30)	**Multi-modality**, (Liver) HCC	Orientation augmentation	20x	1024x1024	-	100-4000	Learned	VGG19	K means clustering	Missing values removed	genes greatest variance selected then WGCNA	Key modules selected by LASSO, top hub gene of 5 modules	No	Cross-validation	67:33	5	-	-	-	-	-	Yes	-
Hou, 2023 (31)	**HGCN**, (Multi - 6) KIRC	-	10x	512x512	-	-	Learned	KimiaNet (CNN)	All	-	Gene set enrichment analysis	Genomic embeddings in functional groups	Hybrid graph convolutional networks (GCNs) and hypergraph convolutional networks (HCNs) to facilitate intra-modal and inter-modal interactions between multimodal graphs	Cross-validation	-	5	-	-	-	Yes	Yes	Yes	Yes
Ji, 2024 (32)	**HGRS**, (Renal) CCRCC	-	20x, 40x	1000x1000	Random	20	Hand crafted	CellProfiler	Overlap of SVM-RFE and LASSO-COX selection	-	WGCNA	Linear regression (top module), top hub genes of module	No	Cross-validation	70:30	5	-	-	-	-	-	Yes	-
Li, 2020 (33)	**DeepHit**, (Breast) -	Augmentation cropping, orientation, colour	-	256x256	-	-	Learned	ResNet - tissue heat map, DCGMM (CNN) - nuclear	LSTM feature encoder	Filter invalid data, normalise and standardise	-	Gene encoder	No	Cross-validation	-	5	-	-	-	-	-	Yes	-
Li, 2021 (34)	**HGPF**, (Colon) Adenocarcinoma	-	-	1000x1000	Random	20	Hand crafted	CellProfiler	Overlap of SVM-RFE and LASSO-COX selection	Normalised	WGCNA	Gene module most associated pathology features	No	Cross-validation	70:30	10	-	-	-	-	-	-	-
Li, 2022 (35)	**HFBSurv**, (Multi -10) Breast	-	-	1000x1000	-	10	Hand crafted	CellProfiler	RandomForestSRC	missing values removed, discretised gene expression and CAN.	-	RandomForestSRC	Modality-specific and cross-modality attentional factorized bilinear modules	Cross-validation	80:20	5	-	-	-	-	Yes	-	Yes
Liu, 2023 (36)	**MGCT**, (Multi - 5) All	CLAM tissue segmentation	20x	256x256	-	-	Learned	ResNet50 (ImageNet)	All	-	SNN	All	Mutual-guided cross-modality attention mechanism	Cross-validation	80:20	5 (Monte Carlo)	-	-	-	-	Yes	-	Yes
Liu, 2024 (37)	**IntraSA-InterCA**, (Breast) -	-	40x	1000x1000	Highest image density patches per WSI	10	Hand crafted	CellProfiler	Fselector	Categorise into under, over or baseline expression	-	Fselector	Intra- and inter-modality attention mechanisms	Cross-validation	80:20	5	-	-	-	-	-	Yes	-
Lv, 2021 (38)	**PG-TFNet**, (Colorectum) -	-	1x, 5x, 20x	512x512	Expert ROI annotation	-	Learned	ResNet based tribranch module for features at each scale, then transformer-based fusion for WSI vector	All	-	-	Differential gene expression by negative binomial distribution (DESeq)	Cross-modality transformer fusion	Cross-validation	90:10	10	-	-	-	-	-	-	-
Lv, 2023 (39)	**Trans-Surv**, (Colorectum) -	-	Thumbnail, 5x, 20x	392x392 (5x), 224x224 (20x)	Random sampling of expert annotated ROI	-	Learned	Multiple CNNs (mutltiscale + Patch tissue classifier)	All	CNV estimated by GISTIC2 method and then categories in 1 of 5 groups	-	Overlap of DESeq, edgeR, limma selection	Cross-attention transformer fusion	Cross-validation	80:20	5	-	-	-	-	-	-	-
Ning, 2020 (40)	**Gene+His***, (Renal) CCRCC	Stain normalisation	-	128x128	-	150 (av.)	Learned	CNN	Block filtering post-pruning search (BFPS) algorithm	Transformed from read counts to normalised fragments per kilobase million.	Selection of most variant 8000 (to reduce noise), then WGCNA on these genes	Block filtering post-pruning search (BFPS) algorithm	Cross-attention fusion model	Cross-validation	67:33	10	-	-	-	Yes	-	-	-
Ning, 2023 (41)	**McLR Framework**, (Multi - 3) LIHC	Stain normalisation	-	512x512	Eliminate if <50% tissue coverage	-	Hand crafted	Parameter free threshold adjacency statistics	PCA	-	-	PCA	Ranking and regression constraints for cross-modal learning	Cross-validation	-	10	-	-	-	-	Yes	Yes	Yes

Perez-Herrera, 2024 (42)	**Model 3,** (Breast) -	Stain normalisation (Reinhard method)	-	512x512	Expert annotations	-	Learned	UNet-VGG16 to predict percentage of defined tissue classes	% tumour, stroma, necrosis	Transformed	Stochastic gradient descent classifier (SNP), AdaBoost (expression), Decision tree (CNV)	Top 100 frequently mutated genes (CNV and CNP), top 30 known differentialy expressed genes in BC	No	Unclear	90:10	-	-	-	-	-		-	Yes
Qiu, 2024 (43)	**DDM-net,** (Brain) Glioma	Otsu algorithm for tissue masks	5x, 10x, 20x	512x512	Patches that contain <30% tissue discarded	-	Learned	ResNet50 (ImageNet) with patch level clustering into 10 phenotypes using k-means (300x1024 dimensional embeddings) condensed by pathology encoders and concatenated to single multiscale patient level feature	-	-	SNN-based genomic encoder	Top 5000 variables with highest variance each omic modality. SVM-RFE selects 162 features fed into SNN	Achieved through dual-space disentanglement via separate variational autoencoders (VAEs), enabling the model to learn shared and modality-specific representations	Cross-validation	-	5	-	-	-	-	-	Yes	-
Shao, 2020 (44)	**OMMFS,** (Multi - 3) LUSC	-	-	5000x5000	Regions defined by experts	4-6	Hand crafted	Extract 10 types nuclear features per patch. Then 10 bin histogram using 5 statistics	Log rank, OMMFS	-	co-expression network analysis, then summarise gene modules by singular value docomposition	Log rank test, OMMFS	Via Ordinal Multi-modal Feature Selection (OMMFS)	Random split data	80:20	N/A	-	-	-	-	Yes	-	Yes
Shao, 2023 (45)	**FAM3L,** (Multi - 3) Multi (KIRP)	-	-	1024x1024	Image density >0.7 (based on percentage of non-white values)	-	Hand crafted	Unsupervised nuclear segmentation, cell level morphological and topological features extracted. Then 10 bin histogram with 5 statistics used to aggreage to image level	-	-	lmQCM	All	Via Hilbert-Schmidt Independence Criterion (HSIC)	Cross-validation	-	5	-	-	-	-	Yes	-	Yes
Shao, 2020 (46)	**M2DP,** (Multi - 3) BRCA	-	-	3000x3000	ROIs chosen by expert	2-8	Hand crafted	Unclear	Unclear - 7 cell level features from extracted nuclei aggregated into 105 dimension feature	-	lmQCM	All	Via Task Relationship Learning	Cross-validation	-	5	-	-	-	-	Yes	Yes	Yes
Shao, 2023 (47)	**IMO-TILS,** (Multi - 3) Breast	-	-	512x512	1) By RGB densitity 2)200 patches with largest TIL ratio	2-8	Learned	Unet++ (segment tILS and tumour tissue), ResNet-101, KNN	Graph attention network pooling	-	lmQCM	Concrete autoencoder (miRNa and mRNA)	Achieved through a deep generalized canonical correlation analysis (DGCCA) with an attention mechanism	Cross-validation	80:20	5	-	-	-	-	Yes	Yes	Yes
Steyaert, 2023 (48)	**Late fusion,** (Brain - Multi) Adult Glioblastoma	Stain augmentation, otsu tissue segmentation	20	224x224	Random	100	Learned	ResNet50 CNN	All	Missing values removed, Gene counts normalised by log and z score transformation, batch effect adjustment (Combat-Seq)	Multi-layer perceptron	-	No	Cross-validation	80:20	10	0.778	x	Yes	-	Yes	Yes	Yes
Subramanian, 2021 (49)	**GCCA,** (Breast) -	Nuclei segmentations from another study	-	2000 x2000	Random	25	Hand crafted	CellProfiler	Averaged across patches	-	-	Selection of most variant genes and correspinding z-scores using coefficient of variation of log transformed expression values,then use of STRING database to capture prior knowlegdge and assign interaction weights	Via sparse canonical correlation analysis (SCCA) to capture both intra-modality and inter-modality correlations	Cross-validation	70:30	5	-	-	Yes	-	-	Yes	-
Subramanian, 2024 (50)	**SCCA,** (Breast) -	Nuclei segmentation masks generated	-	2000 x2000	Random	25	Hand crafted	CellProfiler	5 bin histgram for each of the 215 features	-	-	Most variant genes using coefficient variation of log2 transformed expression values, selecting top 1000 genes.	Via a probabilistic graphical model	Cross-validation	75:25	5	-	-	Yes	-	-	Yes	-
Sun, 2018 (51)	**GPMKL,** (Breast) -	-	40x	1000x1000	Densest tiles per WSI	10	Hand crafted	CellProfiler	Fselector	Gene expression normalised and discretised into under, over or baseline expression. Gene methy.ation and protein expression normalised to z-score.	-	F-selector (information gain ratio measure)	Using multiple kernel learning techniques	Cross-validation	80:20	10	-	-	-	-	-	Yes	-
Tan, 2022 (52)	**MultiCoFusion,** (Brain) GBMLGG	-	-	512x512	Cropped from 1024x1024 expert chosen ROI	-	Learned	ResNet-152 (ImageNet)	All	-	Sparse graph convultional neural network (SGCN)	-	Through multi-task correlation learning	Cross-validation	80:20	15 randomised assignme	-	-	Yes	-	-	Yes	-
Vale-Silva, 2021 (53)	**MultiSurv,** (Multi - 33) Multi	Otsu algorthm for tissue segmentation, Orientation and colour augmentation	-	299x299	-	-	Learned	ResNet50 CNN (ImageNet)	All	CNV categorised into loss, gain or neutral, batch normalisation for omic and clinical modalities	FC CNN	All	No	Bootstrap	90:10	?	-	-	-	Yes	Yes	Yes	Yes
Vollmer, 2024 (54)	**Random Survival Forest,** (Oral) SCC	Colour normalization	40x	1024x1024	10 Highest density patches (ref 19)	-	Hand crafted	CellProfiler	-	Gene filtering for missing values and data normalisation steps to adjust for variability in the data	Top 200 diffferentially expressed	PCA and ElasticNet	No	Cross-validation	-	5	-	-	Yes	-	-	Yes	-

Wang, 2021 (55)	**GPDBN**, (Breast) -	-	-	1000x1000	10 Highest density patches (ref 19)	-	Hand crafted	CellProfiler	Fselector	Normalisation with Z score, discretised into under, over, baseline.	-	Fselector	Bilinear pooling, enabling interaction between image and genomic features	Cross-validation	80:20	5	-	-	Yes	-	-	Yes	-
Wang, 2023 (56)	**HC-MAE**, (Multi - 6) LGG	CLAM tissue segmentation	-	16x16, 256x256, 4096x4096	If >1 WSI per patient - randomly select 1. CLAM to select sub-regions with high diagnostic value	-	Learned	pre-trained Vision Transformer-based Masked Autoencoders	All	Remove genes with zero variance, differential gene expression analysis	MLP	Remove genes with zero variance, differential gene expression analysis, then Random survival forest to select based on feature importance	Hierarchical cross-attention mechanisms integrating histopathological image representations and multi-omics data	Cross-validation	-	5	-	-	-	-	Yes	Yes	Yes
Wei, 2023 (57)	**MultiDeepCox-SC,** (Stomach) -	Colour normalisation, also data augmentation (orientation)	selection of c	512x512	1/9 512x512 from larger 1536x1536 patch (chosen from whole image due to high nuclei density)	-	Hand crafted	CellProfiler	SIS + LASSO	-	Removed or selected based on variance	Intersection of values selected by Sure independent screening + LASSO	No	Cross-validation	-	10	-	-	-	Yes	-	Yes	-
Wu, 2023 (58)	**CAMR**, (Multi - 3) LGG	-	-	1000x1000	Selected by highest RGB image density	10	Hand crafted	CellProfiler	RandomForestSRC	Categorisation into under, over, baseline expression	-	RandomForestSRC	Adversarial alignment and cross-modality fusion modules	Cross-validation	80:20	5	-	-	-	-	Yes	-	Yes
Xie, 2024 (59)	**GaCaMML,** (Stomach)	Tissue segmentation	40x	256x256	-	>10,000	Learned	ResNet50 (ImageNet)	All	-	Two layer FC CNN with ELU activation function	231 genes from 9 chosen oncology pathways	Cross-modal attention mechanism that aligns and fuses features from histopathological images and gene expression data. Additionally, Multiple Instance Learning (MIL) is applied to handle the variable sizes of the image patches and to learn spatially and contextually relevant information from them.	Cross-validation	80:20	5	0.573	x	Yes	-	-	-	-
Zeng, 2020 (60)	**Multi-omics model,** (Head & Neck) SCC	Tissue segmentation	-	1000x1000	Random	20	Hand crafted	CellProfiler	All	Normalisation transcriptomic data	-	100 most common somatic mutation, 100 most DEG (transcriptoimic), All proteomic	No	Cross-validation	50:50	10	-	-	-	Yes	-	Yes	-
Zeng, 2021 (61)	**Multi-omics model**, (Ovary) HGSOC	-	-	1000x1000	Random	60	Hand crafted	CellProfiler	All	-	-	100 most common somatic mutation, 100 most DEG, all proteomic features	No	Cross-validation	50:50	5	-	-	-	Yes	-	Yes	-
Zhan, 2021 (62)	**Cox-nnet,** (Liver) HCC	Patches processed to rescale RGB values to selected slide, tissue folds removed	-	1000x1000	Expert annotated ROI, 10 densest patches within region by RGB	10	Hand crafted	CellProfiler	All	Normalised into RPKM by TCGA-Assembler	-	-	Two-stage Cox-nnet model that integrates hidden features from separate Cox-nnet models for histopathology and transcriptomics	Cross-validation	80:20	5	-	-	-	-	-	Yes	-
Zhang, 2020 (63)	**HI-MKL**, (Brain) Glioblastoma	-	20	1024x1024	Highest nuclear density	20	Hand crafted	CellProfiler	Mutual feature selection methods (mRMR)	Normalised by calculating z index of all variables	-	Mutual feature selection methods (mRMR)	Using multiple kernel learning techniques	Cross-validation	-	10	-	-	Yes	-	-	Yes	-
Zhao, 2023 (64)	**Ada-RSIS**, (Multi - 3) UCEC	-	-	-	-	-	Hand crafted	Parameter-free threshold adjacency statistics	Principal component analysis	-	-	Principal component analysis	Joint learning of sharable and individual subspaces from multi-modality data, incorporating intra-modality complementarity and inter-modality incoherence.	Cross-validation	-	10	0.672	x	-	-	Yes	Yes	Yes
Zheng, 2024 (65)	**FSM**, (Lung - Multi) LUAD	Tissue segmentation	-	-	-	-	Learned	CNN (pretrained)	-	Normalisation of gene counts (EdgeR Biconductor package), filtering out of duplicate and invariant genes, batch correction (ComBat)	FC CNN	Gene signatures assiciated with B cell populations,	Graph attention mechanism that integrates embeddings from pathology images and gene expression data	Cross-validation	-	5	0.579 (c stat)	0.006 (StD)	-	-	-	Yes	-
Zhou, 2023 (66)	**CMTA**, (Multi - 5) GBMLGG	CLAM tissue segmentation	40	512x512	-	-	Learned	ResNet-50 (ImageNet)	-	-	Grouped into functional 6 oncology categories then fed in to FC CNN	-	Cross-modal attention module that facilitates interactions between pathology images and genomic profiles	Cross-validation	-	5	-	-	-	-	Yes	-	Yes
Zhou, 2023 (67)	(Colon) Adenocarcinoma	Colour normalisation (Macenko), backgroud removed	20	512x512	>50% overlap with expert generated tumour annotation mask	-	Learned	Inception V3 (ImageNet)	All	-	-	207 genes from 11 canonical pathways, filtered to remove genes not frequenctly mutated in TCGA-COAD	No	Cross-validation	70:30	100 (Monte carlo)	Unclear	-	-	-	-	Yes	-
Zhou, 2024 (68)	**MSEN**, (Multi - 5) GBM-LGG	-	-	4096x4096	Removal of patches not meeting crieria for HIPT model	-	Learned	HIPT (Hierarchical image pyramid transformer)	All	-	-	Copy number frequency, mutation frequency >10%/5% repsectively. Genes with relevance by Molecular Sigantures Database categorised into 6 functional gene classes.	Cross-modality attention mechanism	Cross-validation	80:20	5	-	-	-	-	Yes	Yes	Yes
Zhu, 2023 (69)	**SAMMS**, (Multi - 2) LGG	ScoreTiler tool for image segmentation	20	4096x4096	-	-	Learned	SAM (pretrained)	ElasticNet (most salient across both modalities)	z score normalisation across datasets	-	2000 Features with most significant variance across modalities, then ElasticNet (most salient across both modalities)	Achieved through a foundation model that learns joint representations across modalities	Cross-validation	-	5	-	-	-	Yes	Yes	Yes	Yes

1.5.1 Figure S1. Modelling method trends over time

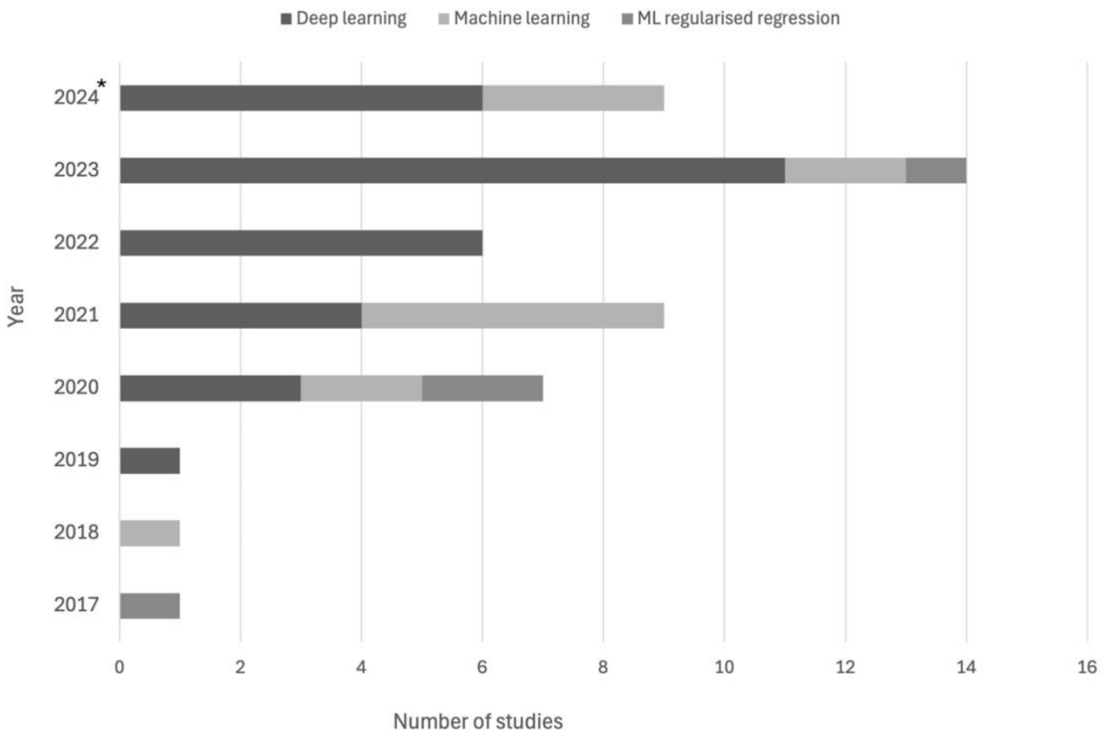

Figure S1. Prediction modelling trends.

This chart highlights the use of different modelling approaches used by the studies of this review, and presents their use over time. *Searches conducted up to August 2024. The apparent drop in overall studies for 2024 is most likely to the mid-year date of the searches. Studies were assigned to one of three approaches: ML regularised regression, classical machine learning or deep learning. Categories used are defined in the supplementary section “author definitions”. There is a predominance of deep learning methods in studies of this review, increasing rapidly in number since 2019.

1.5.2 Figure S2. Model performance by cancer dataset

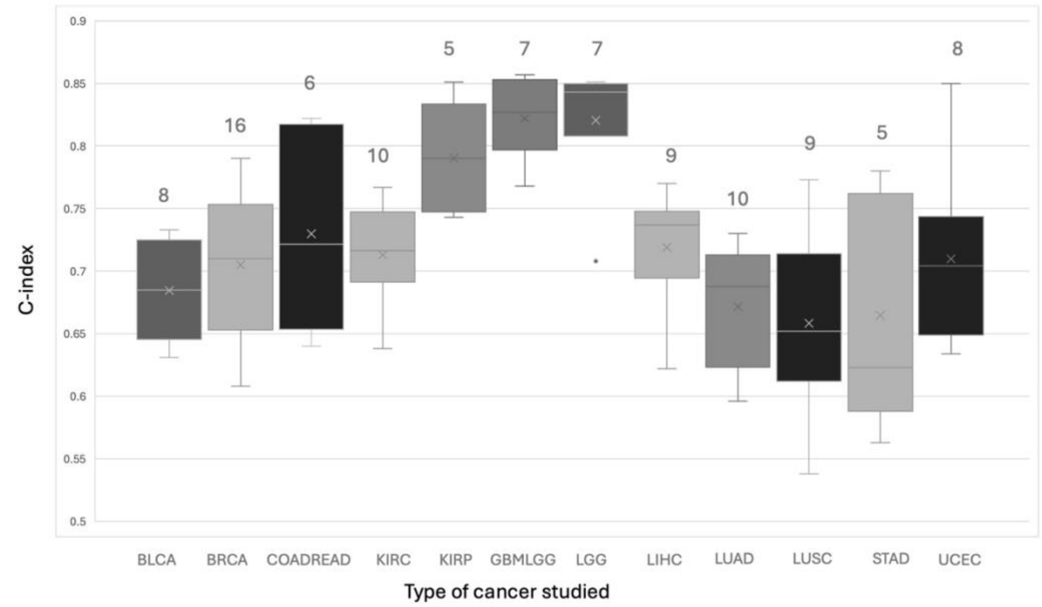

Figure S2. Model performance across cancer types

This comparison was performed for cancer types in which there were more five or more results across the studies in this review. Results from alternate datasets to the one defined as the model of interest for the purposes of this review are also included in this analysis. The number of results included is indicated above the box and whisker plot for each cancer type. Cancer types use the abbreviation nomenclature used by The Cancer Genome Atlas project; BLCA (bladder, urothelial carcinoma)[23,26,35,36,64,66,68,69], BRCA (breast, invasive carcinoma)[23,26,33,35-37,45-47,50,51,55,56,58,66,68], COADREAD (colon and rectum, adenocarcinoma)[23,26,35,38,39,56], KIRC (kidney, renal clear cell carcinoma)[23,26,27,31,35,40,41,44,45,47], KIRP (kidney, renal papillary cell carcinoma)[23,26,35,44,45], GBMLGG (brain, glioblastoma multiforme and lower grade glioma)[27,36,43,48,52,66,68], LGG (brain, lower grade glioma)[23,26,35,56,58,64,69], LIHC (liver, hepatocellular carcinoma)[23,26,30,31,35,41,46,56,62], LUAD (lung, adenocarcinoma)[23,26,31,35,36,41,56,65,66,68], LUSC (lung, squamous cell carcinoma)[23,26,31,35,44,46,47,58,65], STAD (stomach, adenocarcinoma)[23,26,56,57,59], UCEC (Uterine corpus, endometrial carcinoma)[23,26,31,35,36,64,66,68]. Reference numbers link to the reference list of the main document.

Performance is shown to be highest in brain tumours (glioblastoma and low grade glioma) and in renal papillary cell carcinoma. Statistical analysis was unsuitable for these heterogenous studies and this should be regarded as a descriptive comparison only.

1.5.3 Figure S3. Model performance by sample size

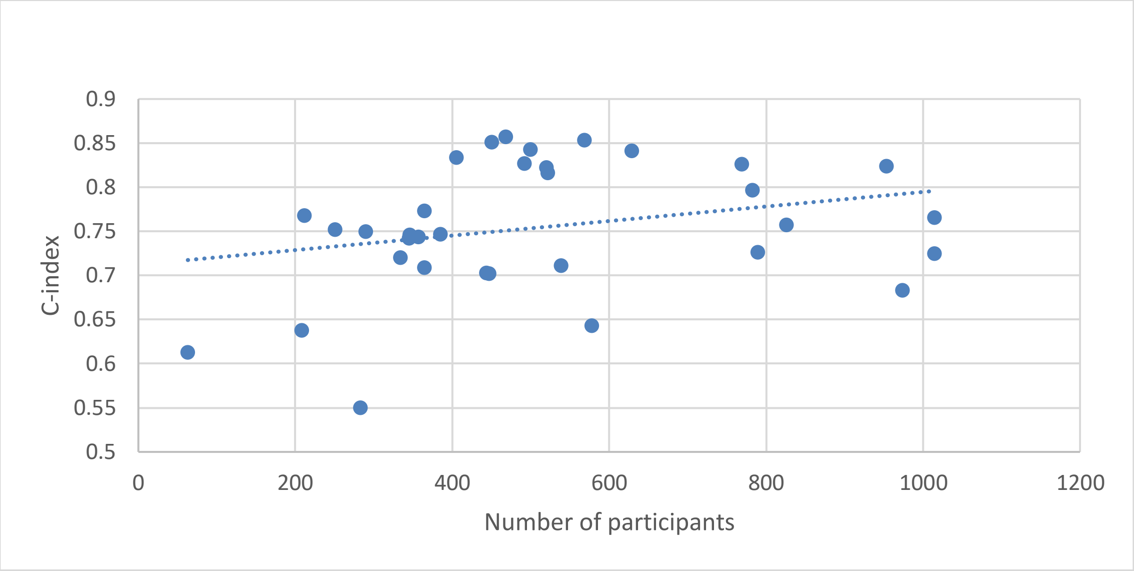

Figure S3. Model performance in relation to participant number. Multimodal model performance of the model of interest compared with number of participants. Thirty nine studies presented results by c-index, however in one study the sample size was unclear, so 38 studies were eligible for inclusion in this comparison. The total number of participants in a study may be greater than presented here due to studies which evaluated their model on multiple datasets separately. There is a trend towards improved performance with increased participant number but studies with sample sizes of ≥400 appear to be sufficient for optimal performance.

1.5.4 Figure S4. Model performance by event rate

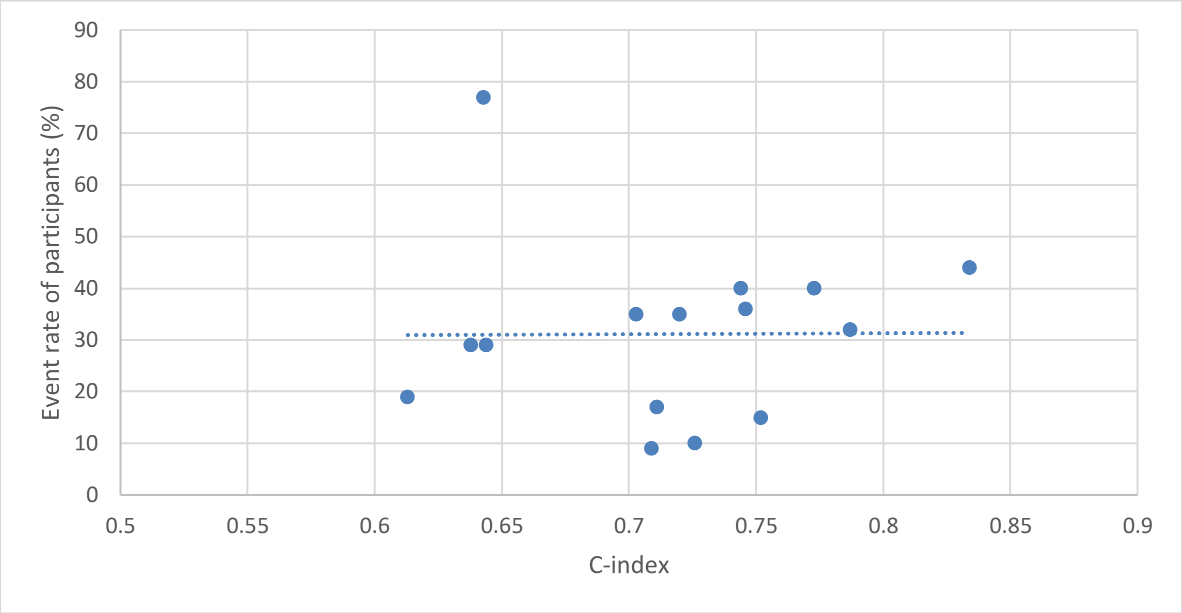

Figure S4.

The relationship between outcome event rate (y-axis) in the studies and performance measured by c-index (x-axis) was explored. Only 15 studies, were eligible for evaluation due to missing values within other studies. There is no apparent relationship between event rate and reported performance in this limited assessment, as demonstrated by the trend line.
